# Optimizing Nav1.7‐Targeted Analgesics: Revealing Off‐Target Effects of Spider Venom‐Derived Peptide Toxins and Engineering Strategies for Improvement

**DOI:** 10.1002/advs.202406656

**Published:** 2024-09-09

**Authors:** Sen Luo, Xi Zhou, Meijing Wu, Gongxin Wang, Li Wang, Xujun Feng, Hang Wu, Ren Luo, Minjuan Lu, Junxian Ju, Wenxing Wang, Lei Yuan, Xiaoqing Luo, Dezheng Peng, Li Yang, Qingfeng Zhang, Minzhi Chen, Songping Liang, Xiuming Dong, Guoliang Hao, Yunxiao Zhang, Zhonghua Liu

**Affiliations:** ^1^ The National and Local Joint Engineering Laboratory of Animal Peptide Drug Development College of Life Sciences Hunan Normal University Changsha 410081 China; ^2^ Peptide and small molecule drug R&D platform Furong Laboratory Hunan Normal University Changsha Hunan 410081 China; ^3^ Institute of Interdisciplinary Studies Hunan Normal University Changsha 410081 China; ^4^ Henan Academy of Innovations in Medical science Institute of Electrophysiology Zhengzhou Henan 450000 China; ^5^ Department of Research Scope Research Institute of Electrophysiology Kaifeng 475004 China; ^6^ Key Laboratory of Hunan Province for Advanced Carbon‐based Functional Materials School of Chemistry and Chemical Engineering Hunan Institute of Science and Technology Yueyang Hunan 414006 China

**Keywords:** analgesic, Kv4.2 and Kv4.3, Nav1.7, off‐target, spider venom‐derived peptide toxins

## Abstract

The inhibition of Nav1.7 is a promising strategy for the development of analgesic treatments. Spider venom‐derived peptide toxins are recognized as significant sources of Nav1.7 inhibitors. However, their development has been impeded by limited selectivity. In this study, eight peptide toxins from three distinct spider venom Nav channel families demonstrated robust inhibition of hNav1.7, rKv4.2, and rKv4.3 (rKv4.2/4.3) currents, exhibiting a similar mode of action. The analysis of structure and function relationship revealed a significant overlap in the pharmacophore responsible for inhibiting hNav1.7 and rKv4.2 by HNTX‐III, although Lys25 seems to play a more pivotal role in the inhibition of rKv4.2/4.3. Pharmacophore‐guided rational design is employed for the development of an mGpTx1 analogue, mGpTx1‐SA, which retains its inhibition of hNav1.7 while significantly reducing its inhibition of rKv4.2/4.3 and eliminating cardiotoxicity. Moreover, mGpTx1‐SA demonstrates potent analgesic effects in both inflammatory and neuropathic pain models, accompanied by an improved in vivo safety profile. The results suggest that off‐target inhibition of rKv4.2/4.3 by specific spider peptide toxins targeting hNav1.7 may arise from a conserved binding motif. This insight promises to facilitate the design of hNav1.7‐specific analgesics, aimed at minimizing rKv4.2/4.3 inhibition and associated toxicity, thereby enhancing their suitability for therapeutic applications.

## Graphical abstract

This study reveals that the off‐target Kv4.2/4.3 may be a common action mechanism of Nav1.7 peptide inhibitors from spider venoms. Pharmacophore‐guided rational design is used to engineer an optimized mGpTx1‐SA, which improves Nav1.7 selectivity, reduces side effects, and enhances analgesic efficacy. This underscores the value of considering off‐target effects and provides a strategy for rational Nav1.7 analgesic design with minimal side effects.

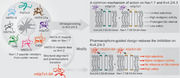

## 1 Introduction

Voltage‐gated sodium (Nav) channel Nav1.7 is preferentially expressed in the peripheral nervous system and is essential for action potential (AP) generation in sensory neurons due to its role in the initial signal amplification, possibly serving as a threshold channel for APs.^[^ ^1^ ^]^ Clinical and genetic evidence shows that Nav1.7 plays an important role in the perception of pain signals.^[^ ^1^, ^2^ ^]^ Accordingly, Nav1.7 is considered as an analgesic target, and pharmacological inhibition of Nav1.7 represents a promising therapeutic strategy for pain relief.^[^ ^3^ ^]^

Venomous animals have evolved numerous cysteine‐rich peptide toxins, many of which are neurotoxins by targeting Nav1.7.^[^ ^4^ ^]^ These venom peptide toxins have been demonstrated to be valuable pharmacological probes and potential drug candidates for Nav1.7.^[^ ^4^, ^5^ ^]^ At present, a variety of Nav1.7 inhibitory peptides derived from spider venoms are known to function as gating modifier toxins (GMTs) and have garnered significant attention in novel analgesics development over the years.^[^ ^6^ ^]^ Notably, the majority of these Nav1.7 inhibitors belong to diverse peptide families of Nav channel‐targeting spider venom toxins (NaSpTx families 1–3) and demonstrate varying degrees of selectivity, particularly within the NaSpTx1 family.^[^ ^5^, ^7^ ^]^ For example, toxins from the NaSpTx family 1 include HNTX‐III, HWTX‐IV, GpTx1, Cl6b, Ca2a, and HNTX‐IV^[^ ^6^, ^8^ ^]^; Pn3a, along with our laboratory's newly identified HNTX‐VIIa, belong to NaSpTx family 2^[^ ^6c^ ^]^; ProTx‐II and GrTx1 are from NaSpTx family 3.^[^ ^6^, ^9^ ^]^ Some of them have been found to exhibit analgesic effects in various rodent pain models and show promising potential in the development of analgesics.^[^ ^6^, ^8^, ^9^, ^10^ ^]^ Recently, these spider venom‐derived peptide toxins have been engineered as template molecules for higher affinity and subtypes selectivity. For instance, engineered analogues of HNTX‐III, GpTx1, HWTX‐IV, and ProTx‐II show ≈1000‐fold selectivity over Nav1.4 and Nav1.5 channels, which are crucial for normal skeletal muscle and cardiac function,^[^ ^8^, ^11^ ^]^ respectively. However, extensive research has shown that the selectivity of peptide toxins is not as high as initially anticipated.^[^ ^12^ ^]^ As more ion channels were examined, it became apparent that peptide toxins previously thought to have high selectivity for Nav subtypes also interact with other types of ion channels.^[^ ^12^ ^]^ This phenomenon may be attributed to the sequence and structural conservation that exists among different types of ion channels.^[^ ^13^ ^]^ For example, GpTx1 was initially identified as a voltage‐gated calcium (Cav) channel Cav3.1 inhibitor;^[^ ^14^ ^]^ ProTx‐I has been discovered to target multiple ion channels, including Nav1.1‐1.8, voltage‐gated potassium (Kv) channel Kv2.1, Cav3.1, Cav3.2, and TRPA1.^[^ ^15^ ^]^ Nav1.7 inhibitors with low selectivity may lead to severe side effects due to off‐target effects on other ion channels or other unknown targets. Therefore, selectivity remains crucial for Nav1.7‐targeted analgesics, as it can mitigate side effects and minimize the loss of peptide toxins targeting Nav1.7 by other non‐Nav1.7 targets, thereby increasing the effective concentration of peptide toxins targeting Nav1.7.

In the present study, our objective is to explore the cross‐activity on ion channels and uncover the underlying mechanism of hNav1.7 peptide inhibitors derived from spider venom. We selected eight peptides from three distinct NaspTx families 1–3, respectively. Our results demonstrate that these peptides robustly inhibit rKv4.2 and rKv4.3 (rKv4.2/4.3) channels. Kv4.2 and Kv4.3, members of the Shal channel subfamily encoded by the *KCND2* and *KCND3* genes, are channels primarily expressed in the nerves and the heart.^[^ ^16^ ^]^ In cardiomyocytes, Kv4.2/4.3‐mediated I_to_ currents are critical for the repolarization of myocardial APs, and a decrease in I_to_ currents may lead to the prolongation of the AP duration.^[^ ^17^ ^]^ Loss‐of‐function or gain‐of‐function mutations can cause various cardiac diseases, including lethal atrial fibrillation^[^ ^18^ ^]^ and ventricular arrhythmias,^[^ ^19^ ^]^ suggesting that inhibition of Kv4.2/4.3 may cause serious cardiac side effects. For example, PaTx1, a specific peptide inhibitor of Kv4.2/4.3, can induce prolongation of the c in mouse electrocardiograms and trigger numerous transient cardiac adverse reactions in mice.^[^ ^20^ ^]^ Hence, the inhibition of Kv4.2/4.3 by these spider venom Nav1.7 peptide toxins poses a challenge to their application in analgesic development. Therefore, based on our understanding of the molecular interaction of these peptide toxins with hNav1.7 and rKv4.2/4.3, we proceeded to engineer the mGpTx1 analogue mGpTx1‐SA, which exhibits significantly enhanced selectivity for hNav1.7 by markedly reducing inhibition of rKv4.2/4.3. As a consequence, mGpTx1‐SA minimizes its impact on cardiotoxicity and demonstrates potent analgesic effects in chronic inflammatory and neuropathic pain models. As Nav1.7 is actively investigated as a target for pain therapeutics, our findings may offer insights for future drug discovery endeavors and rational engineering in the development of analgesic drugs with fewer side effects.

## 2 Results

### 2.1 Spider Venom‐Derived Nav1.7 Inhibitors have High Inhibitory Potency on rKv4.2 and rKv4.3

**Figure 1:**
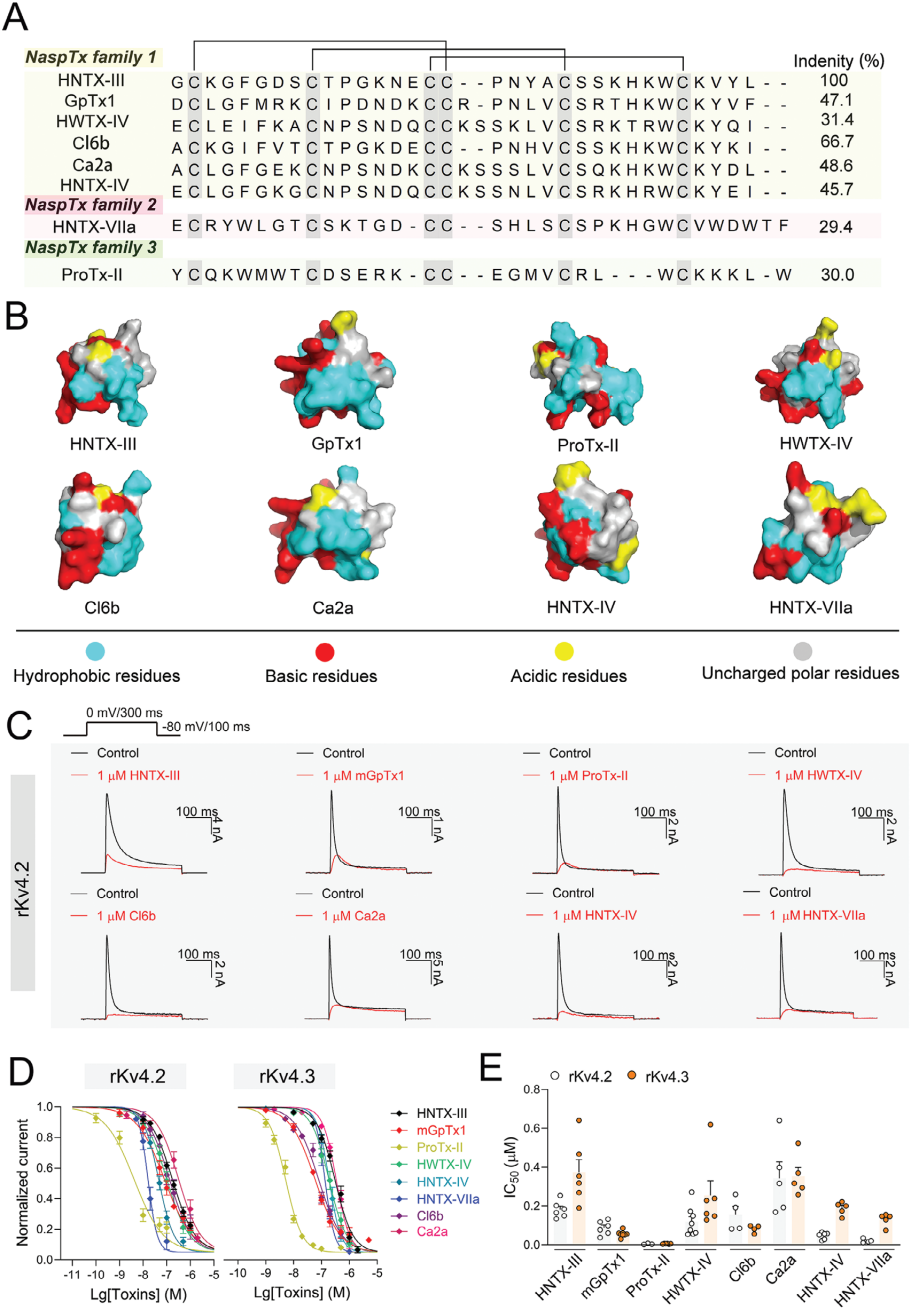
Characterization of the eight spider toxins from NaspTx families 1–3. A) Sequence alignment of HNTX‐III, GpTx1, ProTx‐II, HWTX‐IV, Cl6b, Ca2a, HNTX‐IV, and HNTX‐VIIa by MEGA6.0. B) Surface rendering of structures of HNTX‐III (Protein Databank (PDB) 2JTB), GpTx1, ProTx‐II (PDB 2N9T), HWTX‐IV (PDB 1MB6), Cl6b, Ca2a, HNTX‐IV (PDB 1NIY), HNTX‐VIIa, in which the structures of Cl6b and Ca2a were predicted by I‐TASSER ^[^ ^41^ ^]^; the structures of GpTx1 (PDB AF‐P0DL72‐F1) and HNTX‐VIIa (PDB AF‐D2Y2C3‐F1) were predicted by AlphaFold, respectively. Residues are colored according to their polarity or acidity and alkalinity (cyan, red, yellow and grey represent hydrophobic, basic, acidic, and polar uncharged amino acid residues, respectively). All structures were generated using the molecular graphics program PyMol. C) Representative current traces from HEK293T cells expressing rKv4.2 in the absence (black) and presence (red) of the eight toxins at the concentration of 1 µM, respectively. The inset shows the currents elicited protocol. D) Concentration‐dependent inhibitory curves show the effects of the eight toxins on rKv4.2 or rKv4.3 (n = 3–8). E) The bar diagram shows the IC_50_ values of the eight toxins on rKv4.2 and rKv4.3. Data are represented as mean ± S.E.M.

Of the eight spider venom‐derived peptide toxins mentioned above, seven toxins including HNTX‐III, GpTx1, ProTx‐II, HWTX‐IV, Cl6b, Ca2a and HNTX‐IV have been intensively investigated and are highly active Nav1.7 inhibitors,^[^ ^6^, ^8^, ^9^ ^]^ while HNTX‐VIIa is a newly isolated toxins from the spider *Ornithoctonus hainana* in the present study (**Figure** **1**A). As shown in Figure S1A–D (Supporting Information), the full sequence of HNTX‐VIIa was determined by matrix‐assisted laser desorption/ionization time‐of‐flight mass spectrometry (MALDI–TOF MS/MS) analysis and the venom gland transcriptome cDNA data. It comprises 34 amino acid residues with a molecular mass of 3977.6 Da and its C‐terminal residue Phe34 was amidated. It exhibits high inhibitory activity against hNav1.7 with the half‐maximum inhibitory concentration (IC_50_) of 50.6 ± 4.6 nM (Figure S1E, Supporting Information). HNTX‐VIIa was subsequently used in our further studies after being obtained through chemical synthesis (Figure S1F,G, Supporting Information). The toxins possess a conserved cysteine pattern to form the inhibitor cystine knot (ICK) motif (Figure 1A), and are highly homologous in their 3D structures (Figure 1B). In this study, these eight toxins were chemically synthesized, purchased, or isolated from spider venoms. For GpTx1, mGpTx1, the more potent and selective analogue [Ala^5^, Phe^6^, Leu^26^, Arg^28^] of GpTx1, ^[^ ^8a^ ^]^ was synthesized. We observed that the activity of these peptides was consistent with previous reports^[^ ^6^, ^8^, ^9^ ^]^ (Figure S2A, Supporting Information), and the IC_50_ values of HNTX‐III, mGpTx1, ProTx‐II, HWTX‐IV, Cl6b, Ca2a, HNTX‐IV and HNTX‐VIIa for hNav1.7 were 207.0 ± 57.2 nM, 8.0 ± 1.2 nM, 9.3 ± 1.2 nM, 70.8 ± 22.5 nM, 76.9 ± 4.1 nM, 373.1 ± 86.0 nM, 243.0 ± 42.5 nM, and 80.4 ± 19.6 nM, respectively (Figure S2C, Supporting Information). However, despite excellent in vitro pharmacology, the in vivo analgesic efficacy of these toxins varies, and they were found to be toxic to animals at high doses, leading to adverse effects such as dyskinesia and even death.^[^ ^5^, ^6^, ^9^ ^]^ These findings suggest that they may have uncharacterized off‐target activity. The subtype selectivity of these toxins for Nav channels is clear.^[^ ^6^, ^8^, ^9^ ^]^ To further understand their specificity, we examined whether these toxins affect cardiac Kv channels, which are also the main target of spider venom‐derived peptide toxins and have a homologous structure with Nav channels. As shown in Figure 1C and Figure S2B (Supporting Information), they were indeed active against rKv4.2/4.3, and nearly completely inhibited the peak currents of rKv4.2/4.3 at the concentration of 1 µM. Notably, at this concentration, HNTX‐III, mGpTx1, Cl6b and HNTX‐VIIa had no obvious or only minimal effect on the current traces of other Kv channels, whereas ProTx‐II significantly inhibited the currents of mKv4.1, rKv2.1 and hERG channels, with IC_50_ values of 38.1 ± 5.3 nM, 78.7 ± 9.0 nM, and 29.7 ± 2.8 nM, respectively (Figures S3‐S7, Supporting Information).

Subsequently, we measured the concentration‐dependent curves of HNTX‐III, mGpTx1, ProTx‐II, HWTX‐IV, Cl6b, Ca2a, HNTX‐IV and HNTX‐VIIa to rKv4.2. The IC_50_ values were 162.3 ± 22.6 nM, 80.6 ± 15.8 nM, 4.8 ± 1.8 nM, 58.4 ± 10.5 nM, 114.3 ± 32.3 nM, 267.7 ± 43.8 nM, 51.2 ± 9.9 nM, and 18.0 ± 3.2 nM, respectively (Figure 1D,E). The Hill coefficients of the eight toxins except for HNTX‐VIIa were 1 or close to 1, indicating the 1:1 binding ratio between the toxins and rKv4.2, while that of HNTX‐VIIa was approximately 2, implying that more than one toxin molecules may bind to a channel with possible cooperativity (Table S1, Supporting Information). Similar results were found for rKv4.3, with IC_50_ values of 373.3 ± 65.5 nM, 56.0 ± 4.8 nM, 5.1 ± 0.4 nM, 230.4 ± 44.4 nM, 82.5 ± 11.1 nM, 352.7 ± 46.4 nM, 188.5 ± 14.7 nM, and 130.4 ± 14.5 nM, respectively (Figure 1D,E).

As shown in Figure S8A,B (Supporting Information), the inhibition of rKv4.2/4.3 by the eight toxins was voltage dependent. The current‐voltage (I‐V) curves clearly illustrate that the inhibition ratio of the eight toxins decreases at more depolarized voltages. Specifically, at the depolarization voltage of 100 mV, mGpTx1, ProTx‐II, HWTX‐IV, and HNTX‐VIIa had no effective inhibition on the rKv4.2 currents. Similarly, mGpTx1 also showed no significant inhibition on the rKv4.3 currents at 100 mV (Figure S8B, Supporting Information). This observation is consistent with the effect of toxins on Nav1.7 and supports a model in which toxins bind to the voltage sensor of the channel.^[^ ^6^, ^14^, ^21^ ^]^ Taken together, these results indicate that these toxins may function as gating modifiers, inhibiting rKv4.2/4.3 in a voltage‐dependent or state‐dependent manner. In general, the primary mechanism of GMTs on voltage‐gated ion channels is the regulation of gating kinetics, such as the voltage‐dependence of activation or inactivation. However, apart from ProTx‐II, none of the toxins affected the activation and inactivation curves of Nav1.7.^[^ ^6^, ^8^, ^15^ ^]^ Thus, we then asked whether these toxins altered the gating kinetics of rKv4.2/4.3. As shown in Figure S9A–B and Tables S2 and S3 (Supporting Information), in the presence of sub‐saturation concentrations of the eight toxins, the steady‐state activation and inactivation of rKv4.2 were significantly shifted in the positive direction to varying degrees. Such an effect was also observed in rKv4.3 treated with these toxins, with the exception of Ca2a, which had almost no influence on the steady‐state inactivation of rKv4.3. These data suggest that these toxins may modulate the voltage sensors of rKv4.2/4.3 channels by preferring interaction with the channel's closed or resting state. This was consistent with the action manner of GMTs,^[^ ^20^, ^22^ ^]^ which inhibit rKv4.2/4.3 in a voltage‐dependent manner and alter the channel gating characteristics of activation or inactivation.

### 2.2 rKv4.2 S3‐S4 Linker is Involved in the Binding of Spider Venom‐Derived hNav1.7 Inhibitors

**Figure 2:**
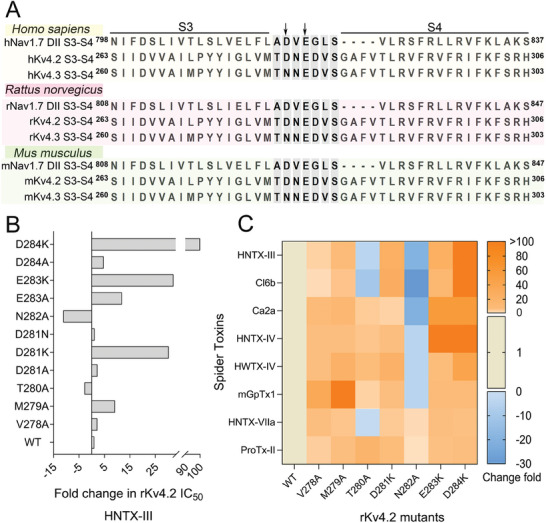
The rKv4.2 S3‐S4 linker is involved in the binding of the eight toxins to rKv4.2. A) Sequence alignment of the S3‐S4 regions of Nav1.7 DII, Kv4.2, and Kv4.3 from human, rat, mouse by MEGA6.0. B) Bar diagram shows the fold changes in IC_50_ values of HNTX‐III for the rKv4.2 mutant channels, compared with that for the WT channel (n = 3–6). **C**) Heat map shows fold changes in IC_50_ values of the eight toxins for rKv4.2 mutant channels, compared with that for the WT channel, and see legend for the change fold (n = 3–8). Data is represented as mean.

Previous studies have reported that the S3‐S4 linker of the voltage‐sensor of DII is critical for the binding of these toxins to Nav1.7.^[^ ^6^, ^8^, ^21^, ^23^ ^]^ We therefore executed a sequence alignment of the S3‐S4 linkers of Nav1.7 DII and Kv4.2/4.3 from human, mouse, and rat, as well as those of other Kv channels. This indicate that the S3‐S4 linkers of Nav1.7 DII and Kv4.2/4.3 share high sequence similarity, regardless of species (**Figure** **2**A), but as for the other Kv channels, no obvious similarity was found (Figure S10, Supporting Information). Their S3–S4 linkers have seven amino acid residues containing at least two acidic ones, indicating negatively charged properties overall. Specifically, D816 and E818 in hNav1.7 correspond to D281 and E283 in rKv4.2, respectively (Figure 2A). These two residues in hNav1.7 are critical for toxin‐channel interaction, and mutations greatly decrease the binding affinity of these toxins.^[^ ^6^, ^7^, ^8^, ^11^, ^21^ ^]^ We therefore examined if the S3‐S4 linker also participated in the toxins‐rKv4.2/4.3 interaction by site‐directed mutagenesis analysis. The single point mutation of rKv4.2 S3‐S4 linker was then constructed, and the changes of HNTX‐III's inhibitory potency on mutant channels were determined (Figure 2B; Figure S11A, Supporting Information). As expected, the two conserved negative residues between rKv4.2 and hNav1.7 (D281 and E283 in rKv4.2), along with the third negative one D284, are of importance, because their mutations to lysine reduced HNTX‐III's inhibition by ≈50‐ to 100‐fold. However, alanine mutations at these sites only reduced HNTX‐III's activity by 2‐ to 12‐fold, suggesting that the negative charge or length of the side chain of the three residues might contribute significantly to the binding of HNTX‐III. For the other four mutations, V278A and M279A slightly decreased, while T280A and N282A slightly enhanced the inhibitory activity of HNTX‐III (Figure 2B; Figure S11A, Supporting Information).

Furthermore, we continued to examine the inhibitory activity of the other seven toxins on rKv4.2 mutant channels. The heatmap shows the contribution of the seven residues of the rKv4.2 S3‐S4 linker to the inhibitory activity of the eight toxins (Figure 2C; Figure S11B, Supporting Information). On one hand, generally speaking, the inhibition changes caused by the mutations had a similar tendency across the eight toxins. For instance, all the three mutations, D281K, E283K, and D284K, resulted in significantly reduced inhibition, although their impacts varied (Tables S4‐S11, Supporting Information). On the other hand, the heatmap revealed some differences among these toxins. For mGpTx1, the most important residue of rKv4.2 for binding is M279, as its mutation to alanine weakened the inhibitory activity by 200‐fold (Figure 2C; Table S5, Supporting Information); the mutation N282A had distinct effects among these toxins, increased activity for HNTX‐VIIa and ProTx‐II, while decreased activity for the other six toxins (Figure 2C; Tables S4‐S11, Supporting Information). These results demonstrated that multiple amino acid residues of rKv4.2 S3‐S4 linker may participate in the binding of these toxins to rKv4.2, although their contributions are not exactly equal. In addition, rKv4.2 and rKv4.3 have only one residue difference in the S3‐S4 linker (D281 in rKv4.2 and N281 in rKv4.3), and D281N mutation did not alter HNTX‐III's activity against rKv4.2 (Figure 2A,B), explaining the similar inhibitory potency of HNTX‐III on both rK4.2 and rKv4.3. Taken together, the S3‐S4 linker is involved in the toxin binding to rKv4.2, although we cannot exclude the possibility of other toxin binding sites.

### 2.3 HNTX‐III has Overlapped Pharmacophore for Targeting hNav1.7 and rKv4.2

**Figure 3:**
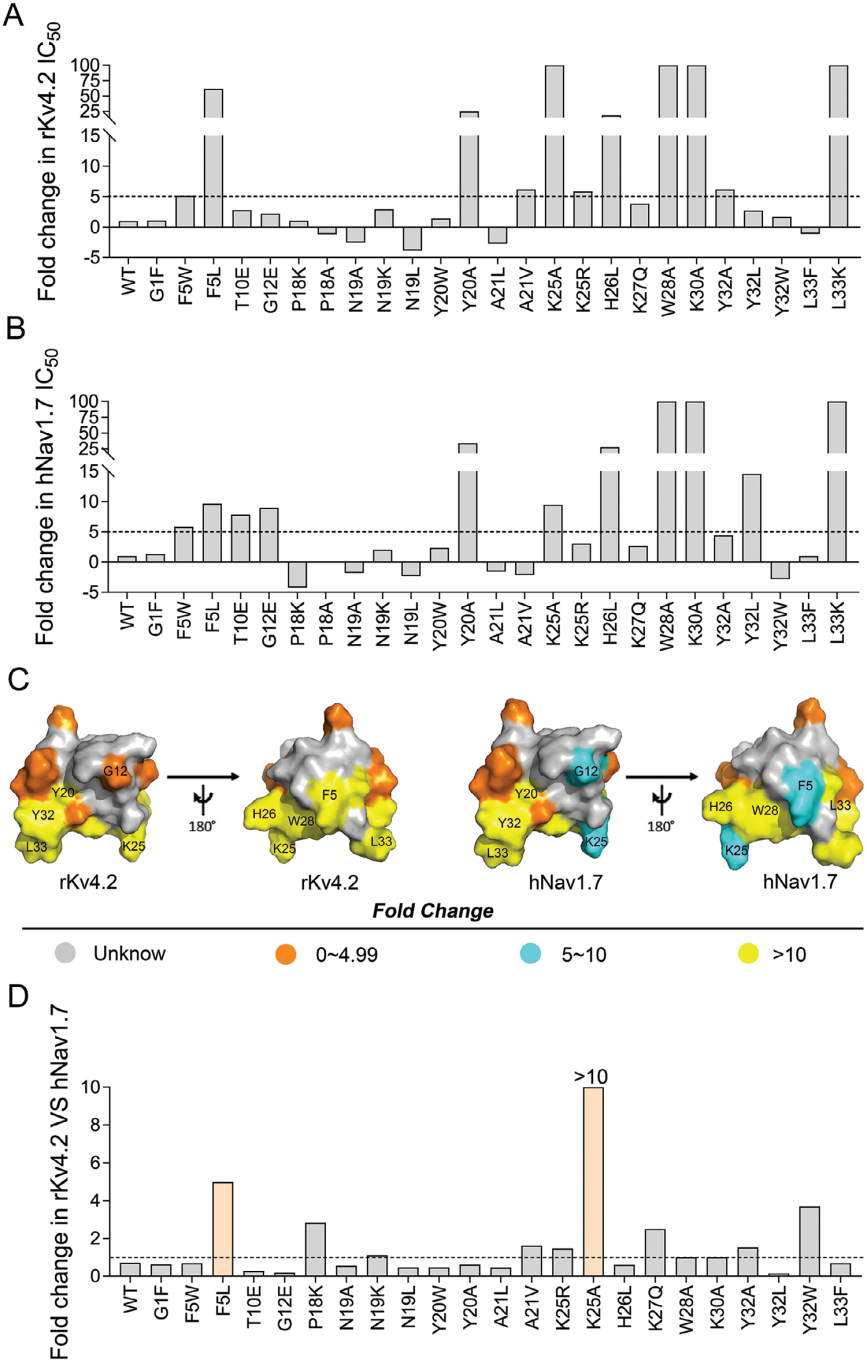
The key residues of HNTX‐III involved in the interaction with rKv4.2. A,B) Bars show the fold changes in IC_50_ values of mutant toxins for rKv4.2 (A, n = 3–8) and hNav1.7 (B, n = 3–8) compared with WT‐HNTX‐III. C) The amino acid residues corresponding to the inhibitory activity for rKv4.2 (left) or hNav1.7 (right) in the surface rendering of NMR structure (PDB code, 2JTB) of HNTX‐III. The residues are colored according to their potency changes, and the data for hNav1.7 are previously reported.^[^ ^11c^ ^]^ D) Bars show the fold changes in IC_50_ values of HNTX‐III mutants for rKv4.2 compared with that for hNav1.7. Data is presented as the mean.

Molecular surface analysis revealed that the eight toxins share common characteristics of the amphiphilic bioactive surfaces, of which one mainly contains basic residues, forming a basic patch on the surfaces of toxins, while the other bioactive surfaces cluster hydrophobic residues, forming a hydrophobic patch (Figure 1B). The residues of the amphiphilic bioactive surface are necessary to determine the high affinity and selectivity for binding to Nav1.7.^[^ ^6^, ^8^, ^11^, ^24^ ^]^ Moreover, the rKv4.2 S3‐S4 linker, which is similar to hNav1.7 DII S3‐S4 in sequence, has also been proven to play an important role in the binding of these toxins to rKv4.2 (Figure 2C). We hypothesized that these toxins might bind to rKv4.2 in a mode similar to hNav1.7. To verify this hypothesis, we chose HNTX‐III as an example to evaluate the structure‐activity relationship of these toxins acting on rKv4.2. According to our previous report on the key amino acid residues for HNTX‐III inhibiting hNav1.7,^[^ ^11c^ ^]^ we prepared a series of mutations of HNTX‐III to explore the potential key residues for the inhibition of rKv4.2. As shown in **Figure** **3** and Table S12 (Supporting Information), mutations of the positively charged amino acid residues in HNTX‐III resulted in a substantial decrease in inhibitory activity, indicating their essential role in the interaction between HNTX‐III and rKv4.2. Specifically, the three mutations K25A, H26L, and K30A significantly reduced the inhibitory effect of the toxin on rKv4.2 by ≈9‐ to 100‐fold, and all of these residues are localized in the basic patch of HNTX‐III (Figure 3C). The mutations of hydrophobic amino acid in the hydrophobic patch, F5L, F5W, Y20A, W28A, Y32A, and L33K, exhibited dramatically decreased inhibition for rKv4.2 (Figure 3A,C). Conversely, when some polar amino acid residues were substituted by hydrophobic residues, such as P18A, N19A, N19L, and A21L, their activities were slightly increased (Figure 3A). Interestingly, our previous study revealed that these mutants also significantly reduced the inhibition of hNav1.7 (Figure 3B),^[^ ^11c^ ^]^ and the N19A, N19L, and A21L mutants also showed increased affinity for hNav1.7 (Figure 3C; Table S12, Supporting Information). As we previously reported,^[^ ^11c^ ^]^ the HNTX‐III potent analogue HNTX‐III‐H4 inhibits hNav1.7 currents with a 30‐fold improved potency and the IC_50_ value is 7.0 ± 1.0 nM. In the present study, we observed that HNTX‐III‐H4 also exhibited increased affinity for rKv4.2/4.3 with 28‐fold and 45‐fold improved potency, respectively. The IC_50_ values for rKv4.2/4.3 were 4.2 ± 0.5 nM and 21.7 ± 2.7 nM, respectively (Figure S12A,B, Supporting Information). Together, these results suggest that the pharmacophores of HNTX‐III targeting hNav1.7 and rKv4.2 are almost overlapped.

### 2.4 Engineering an Analogue of mGpTx1 (mGpTx1‐SA) with Significantly Increased hNav1.7 Selectivity over rKv4.2/4.3

While using HNTX‐III as a paradigm to explore the off‐target mechanism of rKv4.2, we tried to engineer HNTX‐III analogues to obtain optimized peptides with low rKv4.2/4.3 activity and high hNav1.7 activity. Comparing the IC_50_ values of HNTX‐III analogues on rKv4.2 with the ones on hNav1.7 published in our previous study^[^ ^11c^ ^]^ or in this study, we found a 3 to 5‐fold changes in selectivity following the mutations of F5L and Y32W, while the mutant K25A almost lost its inhibition of rKv4.2 and its activity against hNav1.7 only decreased by 8‐fold (Figure 3; Table S12, Supporting Information), suggesting that this residue of HNTX‐III may be more important for rKv4.2 binding. Next, we introduced K25A into the HNTX‐III analogue HNTX‐III‐H4, and its inhibition of rKv4.2 indeed disappeared, but its inhibition of hNav1.7 also decreased to 7.0 ± 0.3 µM (≈1000‐fold) (Figure S13A,B, Supporting Information). Meanwhile, HNTX‐III‐H4‐K25A showed low refolding efficiency (Figure S13C, Supporting Information), suggesting that HNTX‐III‐H4 may not be a suitable template.

**Figure 4:**
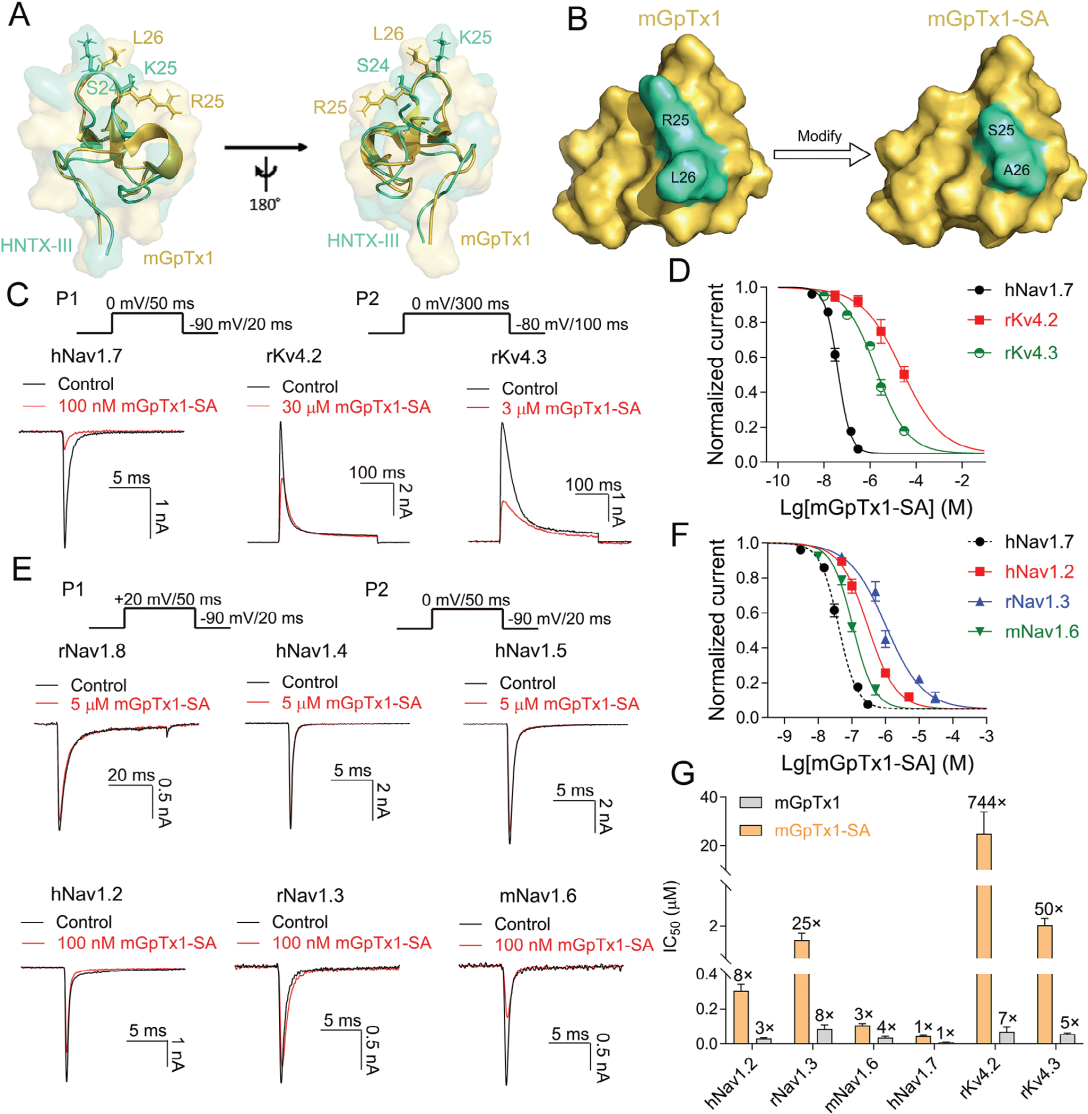
Analysis of the selectivity of mGpTx1‐SA. A) Comparison of the NMR structures of HNTX‐III (yellow‐orange) and mGpTx1 (green‐cyan) superimposed over the disulfide bonds by PyMol. B) Surface rendering of the structures of mGpTx1 and mGpTx1‐SA. The mutated sites are highlighted in green‐cyan. C) Representative current traces from HEK293T cells expressing hNav1.7 (left, n = 5), rKv4.2 (middle, n = 3), and rKv4.3 (right, n = 5) in the absence and presence of mGpTx1‐SA, respectively. The currents of hNv1.7 were elicited by protocol P1, and rKv4.2/4.3 currents were elicited by protocol P2. D) Concentration‐dependent inhibitory curves show the effect of mGpTx1‐SA on hNav1.7 (black, n = 5), rKv4.2 (red, n = 3), and rKv4.3 (green, n = 5). E) Representative current traces of hNav1.2, rNav1.3, hNav1.4, hNav1.5, mNav1.6, and rNav1.8 in the absence (black) and presence (red) of mGpTx1‐SA, respectively (n = 4–6). The currents of Nav1.8 were elicited by protocol P1, and Nav1.2‐1.6 currents were elicited by protocol P2. F) Concentration‐dependent inhibitory curves show the effect of mGpTx1‐SA on hNav1.7 (black, n = 4), hNav1.2 (red, n = 4), rNav1.3 (blue, n = 6) and hNav1.6 (green, n = 5). G) Bars show the IC_50_ values of mGpTx1‐SA and mGpTx1 for hNav1.2, rNav1.3, mNav1.6, rKv4.2 and rKv4.3. The numbers above the bars show the fold changes in IC_50_ values between the indicated channels and hNav1.7 (n = 3–6). Data are presented as the mean ± S.E.M.

Therefore, a new toxin template was needed for subsequent toxin remodeling, and mGpTx1 attracted our attention. The main reasons are as follows: 1) mGpTx1 is an engineered analogue of GpTx1 with optimized activity and selectivity over hNav1.4 and hNav1.5,^[^ ^8a^ ^]^ and has good refolding efficiency; 2) Similar to HNTX‐III, mGpTx1 also binds to the S3‐S4 linker of rKv4.2; 3) The tertiary structures of mGpTx1 and HNTX‐III are extremely similar (**Figure** **4**A). Combining structure and sequence alignment data, we speculated that R25 or L26 of mGpTx1 may correspond spatially to K25 of HNTX‐III and may be crucial for the inhibition of rKv4.2. Two peptides, namely mGpTx1‐R25A (Figure S13D, Supporting Information) and mGpTx1‐R25S‐L26A (mGpTx1‐SA), were obtained (Figure 4B; Figure S13E, Supporting Information). Compared with mGpTx1, mGpTx1‐R25A decreased the IC_50_ value for hNav1.7 to 1.3 ± 0.1 nM, while that of rKv4.2 also decreased to 57.2 ± 6.2 nM (Figure S13F,G, Supporting Information). Encouragingly, mGpTx1‐SA was found to be potent and selective for hNav1.7 activity, with IC_50_ values of 40.3 ± 3.2 nM for hNav1.7, 30.0 ± 9.0 µM for rKv4.2, and 2.0 ± 0.3 µM for rKv4.3, resulting in more than 744‐fold selectivity over rKv4.2 and approximately 50‐fold selectivity over rKv4.3 (Figure 4C‐G).

Next, we confirmed the selectivity of mGpTx1‐SA. As shown in Figure 4E, mGpTx1‐SA exhibits no effect on the currents of hNav1.4, hNav1.5, rNav1.8. Meanwhile, when compared to its inhibition of hNav1.7, mGpTx1‐SA significantly decreased its potency for hNav1.2, rNav1.3, and a mutant channel of mNav1.6 (Y362S, TTX‐R)^[^ ^25^ ^]^ by 8‐fold, 25‐fold, and 3‐fold, respectively, thereby enhancing its selectivity ratio for hNav1.7 over other Nav channel subtypes (Figure 4E‐G). Furthermore, as shown in Figure S14 (Supporting Information), mGpTx1‐SA (10 µM) had low inhibition on other Kv channel subtypes and on hCav3.1, hCav3.2, and hCav3.3 channels, although native GpTx1 was first identified as a Cav3.1 inhibitor.^[^ ^14^ ^]^

### 2.5 mGpTx1‐SA has Reduced Cardiotoxicity by Removing the Activity Against rKv4.2/4.3

**Figure 5:**
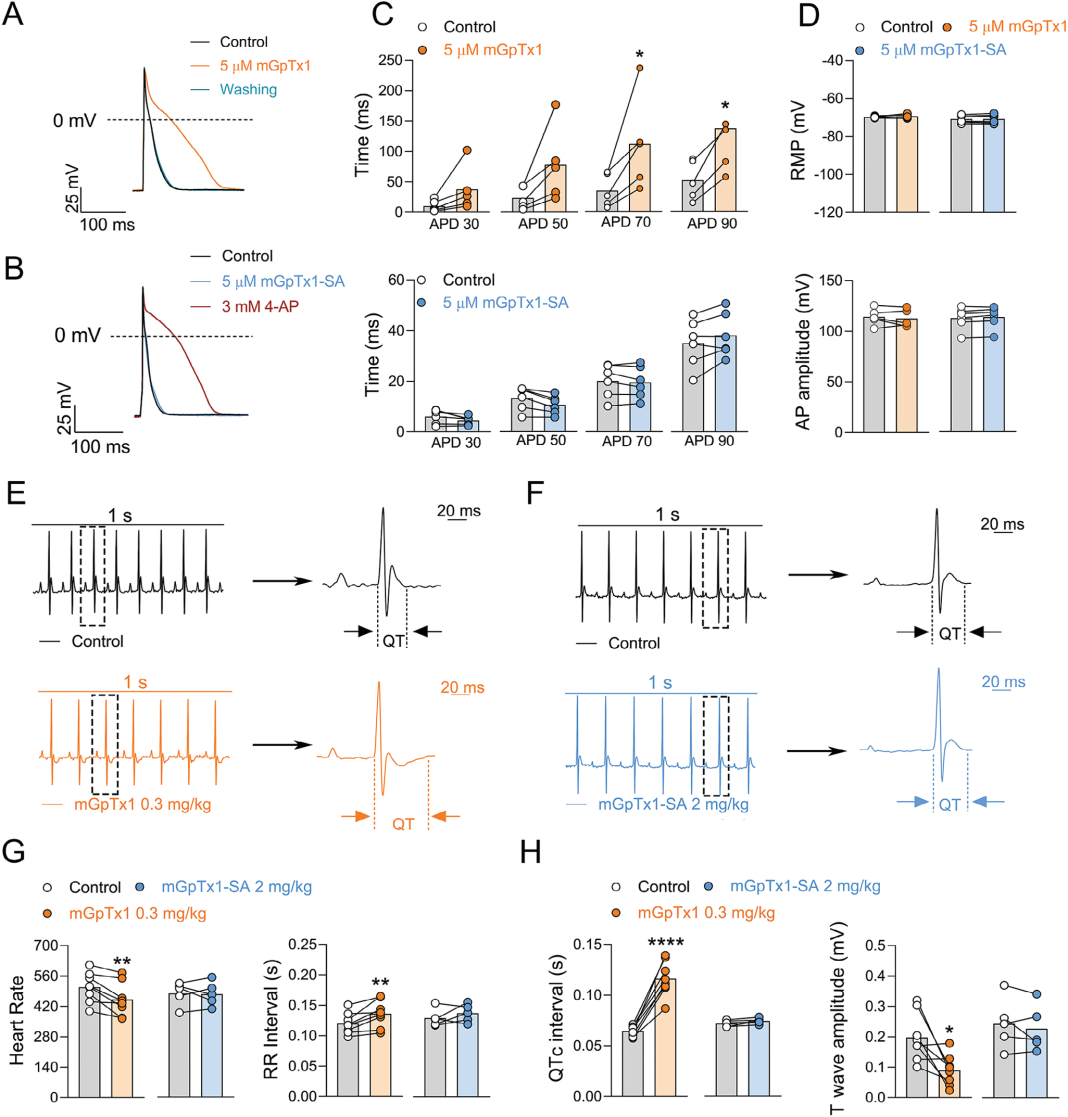
The cardiotoxicity analysis of mGpTx1‐SA. A,B) Representative current traces of action potentials (APs) treated by 5 µM mGpTx1 (orange, n = 5) (A), 5 µM mGpTx1‐SA (blue, n = 6) or 3 mM 4‐AP (brown, n = 6) (B). C) Statistics of the duration of AP (APD) from acutely isolated rat cardiomyocytes treated by 5 µM mGpTx1 (n = 5) or 5 µM mGpTx1‐SA (followed by paired t test, n = 6). D) Statistics of the resting membrane potential (RMP) (top) and AP amplitude (bottom) degrees of APs from acutely isolated rat cardiomyocytes treated by 5 µM mGpTx1 (n = 5) or 5 µM mGpTx1‐SA (followed by paired t test, n = 6). E,F) Representative surface electrocardiogram (ECG) waveforms treated by 0.3 mg kg^−1^ mGpTx1 (orange, n = 8) (E), 2 mg kg^−1^ mGpTx1‐SA (blue, n = 5) (F) or the control (black), which is from lead II surface ECG, recorded from ICR mice by Labchart (AD instrument). The arrows indicate the representative ECG waveforms enlarged from the boxed area of each corresponding trace. G) Heart rate and RR intervals from recordings as in e and f treated by 0.3 mg kg^−1^ mGpTx1 (orange, n = 8), 2 mg kg^−1^ mGpTx1‐SA (blue, n = 5), followed by paired t test. H) T amplitude and QTc intervals from recordings as in e and f treated by 0.3 mg kg^−1^ mGpTx1 (orange, n = 8), 2 mg kg^−1^ mGpTx1‐SA (blue, n = 5), followed by paired t test. Data are represented as mean ± S.E.M. ^*^ *p* < 0.05, ^**^ *p* < 0.01, ^***^ *p* < 0.001, ^****^ *p* < 0.0001 versus control.

As described above, inhibition of rKv4.2/4.3 may affect the APs of cardiomyocytes, leading to severe cardiotoxic effects.^[^ ^20^, ^26^ ^]^ Therefore, we evaluated if mGpTx1 and mGpTx1‐SA affected the APs of cardiomyocytes. As shown in **Figure** **5**A,B, similar to 4‐aminopyridine (4‐AP) which is a rKv4.2/4.3 blocker, 5 µM mGpTx1 significantly prolonged action potential duration (APD) in rat cardiomyocytes. In particularly, it significantly increased APD at 90% repolarization (APD 90) from 51.7 ± 14.6 ms to 137.1 ± 36.3 ms (n = 5, *p* = 0.0289). In contrast, 5 µM mGpTx1‐SA had no effect on APD (Figure 5C). As for AP amplitude and resting membrane potential (RMP), no significant changes were observed in the presence of 5 µM mGpTx1 or mGpTx1‐SA (Figure 5D). Correspondingly, at the concentration of 5 µM, mGpTx1‐SA did not inhibit the Kv and Nav currents in the primary cardiomyocytes (Figure S15, Supporting Information). These results indicate that mGpTx1‐SA lacks the activity of prolonging APD in cardiomyocytes.

Next, surface electrocardiograms (ECGs) were recorded from ICR mice (both male and female). mGpTx1 or mGpTx1‐SA were administered via the tail vein injection. As shown in Figure 5E,F, administration of 0.3 mg kg^−1^ mGpTx1 notably altered the shape of the ECGs and led to a decrease in the number of ECGs recorded over 1 s, whereas 2 mg kg^−1^ mGpTx1‐SA demonstrated no effect on the ECGs. Analysis of the ECGs revealed that 0.3 mg kg^−1^ of mGpTx1 significantly increased the RR interval in mice (control: 0.12 ± 0.01 s; mGpTx1: 0.14 ± 0.01 s; n = 8, *p* = 0.0088), accompanied by a significant decrease in heart rate (control: 508.4 ± 24.5; mGpTx1: 451.6 ± 27.1; n = 8, *p* = 0.0045), whereas mGpTx1‐SA did not affect RR interval (control: 0.13 ± 0.01 s; mGpTx1‐SA: 0.13 ± 0.01 s; n = 5, *p* = 0.3229) and heart rate (control: 478.2 ± 24.3; mGpTx1‐SA: 475.3 ± 24.7; n = 5, *p* = 0.8856) at the high concentration of 2 mg kg^−1^ (Figure 5G). When QT intervals were corrected for heart rate,^[^ ^27^ ^]^ as shown in Figure 5H, the difference between the control and the mGpTx1 treatment animals were also highly significant (n = 8, *p* < 0.001): QTc intervals were 0.07 ± 0.01 s and 0.12 ± 0.01 s, respectively; and T‐wave amplitude was significantly decreased after treatment with 0.3 mg kg^−1^ mGpTx1 (control: 0.20 ± 0.03 mV; mGpTx1: 0.09 ± 0.02 mV; n = 8, *p* = 0.0138). However, in the treatment of 2 mg kg^−1^ mGpTx1‐SA, which did not show the effects on QTc intervals (control: 0.072 ± 0.00 ms; mGpTx1‐SA: 0.074 ± 0.00 ms, n = 5, *p* = 0.085) and T‐wave amplitude (control: 0.24 ± 0.04 ms; mGpTx1‐SA: 0.23 ± 0.03 ms, n = 5, *p* = 0.4032). Overall, these data suggest that inhibiting rKv4.2/4.3 may affect APD in mouse cardiomyocytes and cardiac repolarization, resulting in the prolongation of QTc and the occurrence of arrhythmias.

### 2.6 mGpTx1‐SA Decreases the Membrane Excitability of DRG Neurons and Prevents Mechanical Allodynia in Inflammatory and Neuropathic Pain Models

**Figure 6:**
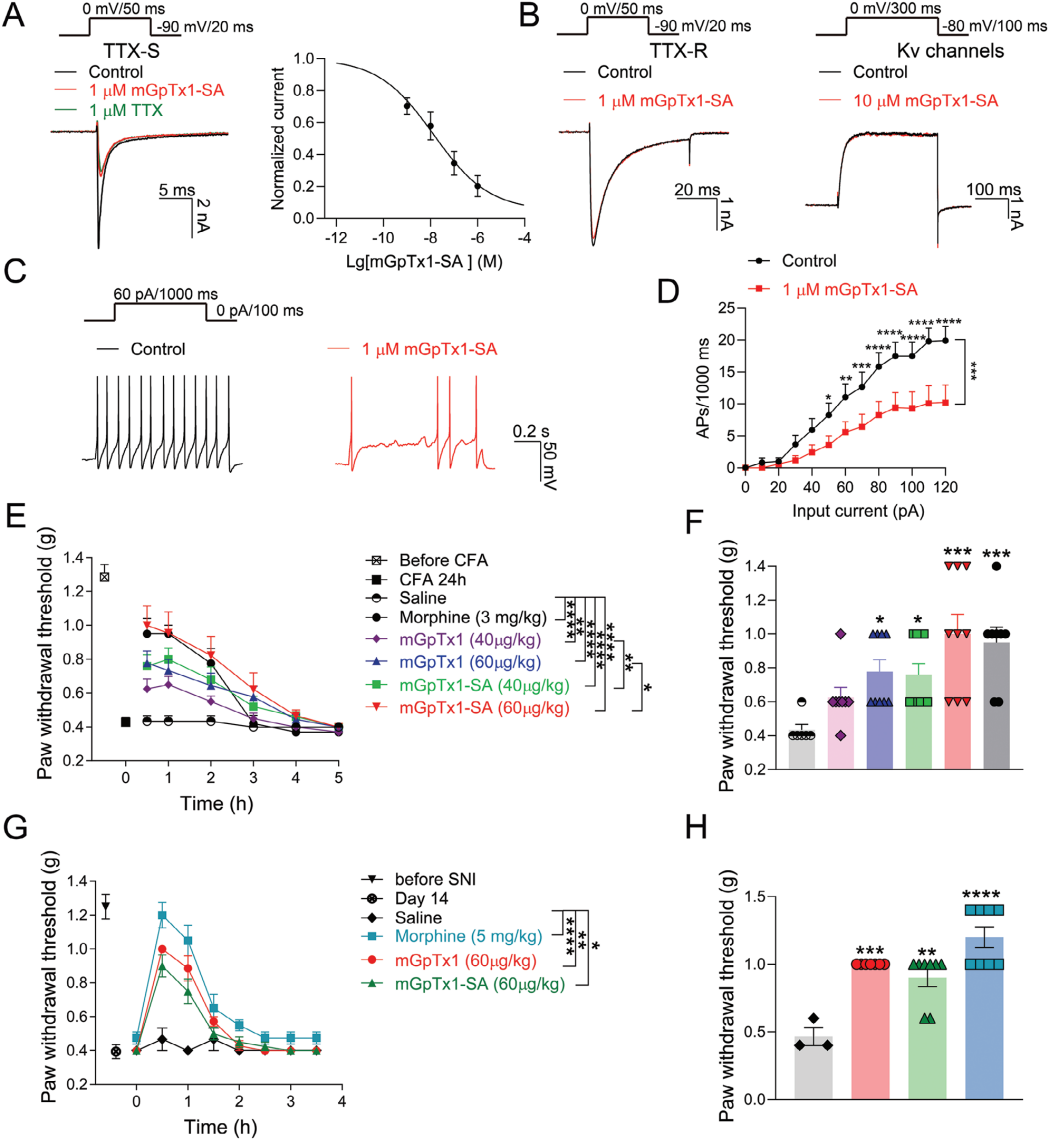
Inhibition of DRG neurons excitability and analgesic effects of mGpTx1‐SA in mice. A) Representative current traces from mouse DRG neurons expressing native TTX‐S Nav channels (left, upper panel shows the voltage protocol, n = 4) treated by 1 µM mGpTx1‐SA (red) or 1 µM TTX (green); and the concentration‐dependent inhibitory curve (right) of mGpTx1‐SA on native TTX‐S Nav channels (n = 4). B) Representative current traces showing the effect of mGpTx1‐SA on mouse DRG TTX‐R Nav currents (left, n = 4) or Kv currents (right, n = 4) elicited by voltage protocols. C) AP traces recorded from a representative mouse DRG neurons before (black) and after (red) the application of 1 µM mGpTx1‐SA. D) Statistics plots show significant decreases in AP spike number in the presence of 1 µM mGpTx1‐SA (n = 14, two‐way repeated measures ANOVA followed by Tukey's multiple comparisons test). E,F) mGpTx1‐SA was more effective than mGpTx1 in reducing the CFA‐induced inflammatory pain (n = 6 to 9 per group). E) Time‐course of analgesic effects of mGpTx1‐SA (i.m.) and mGpTx1 (i.m.) in the CFA test (two‐way repeated measures ANOVA followed by Tukey's multiple comparisons test). F) Statistics of the anti‐nociceptive effect 30 min after the drug injection (one‐way ANOVA followed by Dunnett's post hoc tests). G,H) Analgesic effects of mGpTx1‐SA (i.p.) and mGpTx1 (i.p.) in a saphenous nerve ligation‐induced neuropathic pain model in mice (n = 6 to 9 per group). (G) Time‐course of analgesic effects of mGpTx1‐SA (i.p.) and mGpTx1 (i.p.) (two‐way repeated measures ANOVA followed by Tukey's multiple comparisons test). (H) Statistics of the writhing numbers 30 min after the drug injection (one‐way ANOVA followed by Tukey's post hoc tests). Data are represented as mean ± S.E.M. ^*^ *p* < 0.05, ^**^ *p* < 0.01, ^***^ *p* < 0.001, ^****^ *p* < 0.0001 versus saline or control.

We first assessed the effects of mGpTx1‐SA on the native Na^+^ and K^+^‐mediated currents of dorsal root ganglions (DRG) by voltage‐clamp recording. As shown in **Figure** **6**A and 1 µM mGpTx1‐SA inhibited tetrodotoxin‐sensitive (TTX‐S) Nav currents by 85.0 ± 4.0%, and the IC_50_ was 23.0 ± 11.2 nM. On the contrary, mGpTx1‐SA had no evident effect on tetrodotoxin‐resistant (TTX‐R) Nav channels and Kv channels in DRG neurons (Figure 6B). The effects of mGpTx1‐SA on membrane excitability were also examined on small (<30 µm) DRG neurons from mice by using current‐clamp recordings. With the treatment of 1 µM mGpTx1‐SA, these neurons produced significantly decreased AP firing in response to depolarizing currents (Figure 6C,D). These results indicate that mGpTx1‐SA may reduce the membrane excitability of small DRG neurons.

Chen et al. reported that intrathecal injection of mGpTx1 produces powerful anti‐nociception effects in pain animal models.^[^ ^28^ ^]^ In the present study, we compared the analgesic effects of mGpTx1 and mGpTx1‐SA in pain mouse models. Please note the peptides were administrated by intramuscular/intraperitoneal injection but not intrathecal injection (the former may be more convenient than the latter in the pain treatment). In the CFA‐induced mouse model (both male and female) (Figure 6E,F), mGpTx1‐SA at both concentrations of 40 and 60 µg kg^−1^ could significantly increase the paw withdrawal threshold compared with saline following intramuscular injection, but mGpTx1 only at the concentration of 60 µg kg^−1^ had such effect. It is worth noting that mGpTx1‐SA is more effective than mGpTx1 in this model, and the anti‐allodynic effects of mGpTx1‐SA and mGpTx1 lasted up to 3 h, whereas that of morphine (3 mg kg^−1^) lasted for 2 h.

In addition, we explored the analgesic effects of mGpTx1‐SA on the spared nerve injury (SNI) neuropathic pain model of mice (both male and female). After 14 days of post‐surgery, significant mechanical allodynia was developed on the injured hind paw. As shown in Figure 6G,H, mGpTx1 (60 µg kg^−1^ i.p.), mGpTx1‐SA (60 µg kg^−1^ i.p.) and morphine (5 mg kg^−1^ i.p.) significantly decreased the mechanical allodynia induced by SNI compared with the saline group. Overall, mGpTx1‐SA exhibited excellent analgesic effects in chronic inflammatory and neuropathic pain models.

### 2.7 mGpTx1‐SA Possesses an Improved In Vivo Safety Profile

Considering that mGpTx1‐SA remained attenuated inhibition against some Nav channels, and these channels usually have crucial physical functions. For instance, Nav1.6 is expressed at the nodes of Ranvier in peripheral neurons and plays important roles in motor system;^[^ ^29^ ^]^ the currents of Kv4.2/4.3 are also an important determinant of electrical excitability in colonic smooth muscle cells, and their inhibition may contribute to gastrointestinal motor disorders.^[^ ^30^ ^]^ Therefore, we tested if mGpTx1‐SA affected the motor ability of mice. As illustrated in Figure S16A (Supporting Information), intraperitoneal injection of mGpTx1‐SA at a dose of 2 mg kg^−1^, which is up to 30‐fold higher than the dose (60 µg kg^−1^) exhibiting robust analgesic effects, did not affect the balance time of mice on the rotarod test (n = 5–6, *p* > 0.9999) or the time spent on swimming (n = 5, *p* = 0.8413), compared to the saline group. In addition, mGpTx1‐SA (2 mg kg^−1^) has no effect on small intestinal propulsion compared to the saline group (n = 4, *p* = 0.3429) (Figure S16B, Supporting Information).

Furthermore, we evaluated the effects of mGpTx1‐SA on locomotor activity of mice by open‐field test. As shown in Figure S16C (Supporting Information), mGpTx1‐SA (2 mg kg^−1^) did not influence significantly the mouse exploratory and locomotor behavior because the treated mice traveled in the cage with similar distance and trace to the saline group within 2 min (n = 4, *p* = 0.2000).

## 3 Discussion

In this study, we found that rKv4.2/4.3 are off‐target of some hNav1.7 peptide inhibitors derived from spider venom. This was revealed through an intensive investigation of eight peptide toxins from NaspTx families 1–3. This off‐target activity may lead to severe side effects, such as cardiotoxicity, thereby hindering their development as analgesic agents. Next, by elucidating the action mechanism and pharmacophore of the toxins inhibiting hNav1.7 and rKv4.2/4.3, we proposed a strategy for rationally designing highly selective hNav1.7 peptide inhibitors. As a result, we developed an optimized analogue of mGpTx1(mGpTx1‐SA) which preserves hNav1.7 inhibition while lacking activity against rKv4.2/4.3. Furthermore, mGpTx1‐SA exhibited improved analgesic efficacy and significantly reduced cardiotoxicity and in vivo side effects compared to mGpTx1.

Our results reveal a similar mode of action between the eight toxins inhibiting hNav1.7 and rKv4.2/4.3. They act as gating modifiers for both channel types, obstructing channel activation by directly targeting and impeding the outward movement of the voltage sensor. The voltage sensor of voltage‐gated ion channels emerges as a crucial area of interest in research on interactions with venom‐derived peptide toxins.^[^ ^5^, ^31^ ^]^ On one hand, we discovered that the hNav1.7 DII and rKv4.2/4.3 S3‐S4 linkers exhibit high sequence conservation, a feature not present in other rKv channel subtypes, and they are implicated in the interaction with the eight toxins. The DII S3‐S4 linker forms a part of site 4 in Nav channels, which serves as a prominent binding site for peptide toxins, particularly those from spider venom.^[^ ^5^, ^6^ ^]^ Our rKv4.2 mutagenesis analysis indicates that the S3‐S4 linker of rK4.2/4.3 also plays an important role in the interaction of the eight toxins, although the binding sites other than the S3‐S4 linker may also be involved, as some toxins such as ProTx‐II demonstrate an inhibitory effect on other Kv channels. On the other hand, these toxins exhibit a similar amphipathic surface (Figure 1B), which plays a crucial role in their interaction with hNav1.7 and rKv4.2/4.3. Our results also demonstrate that HNTX‐III utilizes the same pharmacophore to inhibit the activation of hNav1.7 and rKv4.2 (Figure 3A‐C). Given structural and pharmacological similarity of these toxins, we hypothesize that these toxins may interact with hNav1.7 and rKv4.2/4.3 through a similar binding mode. Overall, these results reveal a common mechanism underlying the off‐target effect of spider venom‐derived hNav1.7 peptide toxins by acting on rKv4.2/4.3.

The constrained selectivity of hNav1.7 inhibitors motivates the optimization of existing inhibitors to improve their potency and selectivity. This endeavor has become a popular yet challenging topic in peptide engineering research.^[^ ^8^, ^11^, ^24^ ^]^ Our findings offer novel insights into the advancement of peptide toxin analgesics targeting hNav1.7. By capitalizing on the pharmacological similarities among these toxins and leveraging their spatial conformation homology, we integrated mutagenesis data from HNTX‐III into the sequence of mGpTx1, resulting in the development of the analogue mGpTx1‐SA, which exhibits improved hNav1.7 selectivity by removing/reducing its activity against rKv4.2/4.3. Consistent with the important roles of rKv4.2/4.3 in cardiac APs, eliminating the inhibitory effect on rKv4.2/4.3 could potentially reduce the cardiotoxicity of mGpTx1‐SA and improve its in vivo safety profile (Figure 5; Figure S16, Supporting Information). It's worth noting that Kv4.2/4.3 are also localized at the somatosensory neurons, including DRG (< 30 µm)^[^ ^32^ ^]^ and trigeminal ganglion (TG) neurons (25–34 µm),^[^ ^33^ ^]^ where they regulate the frequency of slow repetitive spike firing and contribute to neuronal excitability. This line of evidence indicates that Kv4.2/4.3 channels are involved in the pain signaling pathway, and decreasing Kv4.2/4.3 currents may induce pain.^[^ ^34^ ^]^ Furthermore, we found that 10 µM mGpTx1‐SA had no significant inhibition on native Kv channels including mKv4.2/4.3 on mouse DRG in this study (Figure 6B). Therefore, it is not surprising that mGpTx1‐SA, with its inhibitory activity removed against Kv4.2/4.3, demonstrates a more potent analgesic effect compared to mGpTx1 in pain models, although it is approximately 4‐fold weaker than mGpTx1 for hNav1.7 inhibition (Figure 6E‐H).

However, mGpTx1‐SA still exhibits some limitations in selectivity, as it retains inhibitory activity against mNav1.6. It is advisable to minimize Nav1.6 activity as much as possible during the optimization of Nav1.7 inhibitors, as this has been reported in few studies.^[^ ^11^, ^24^ ^]^ For example, the modification of ProTx‐II has led to optimized analogs with Nav1.6 inhibition weakened by over 100‐fold.^[^ ^11a^ ^]^ We endeavored to refine mGpTx1‐SA by introducing additional mutations based on published data.^[^ ^11^, ^24^ ^]^ However, this attempt was unsuccessful due to the nearly identical DII S3‐S4 linkers in Nav1.6 and Nav1.7. Nonetheless, we did not observe any motor deficits induced by mGpTx1‐SA at the concentration (2 mg kg^−1^), which exceeds the “therapeutic” dose (60 µg kg^−1^) by over 30‐fold. A possible explanation is that a sufficiently high concentration is required to suppress Nav1.6 to induce motor disorders.^[^ ^35^ ^]^ More interestingly, mGpTx1‐SA might exhibit a stronger analgesic effect than ProTx‐II analogues, given that the “therapeutic” dose for the latter, administered via intramuscular injection, exceeds 2 mg kg^−1^ in animal pain models.^[^ ^11a^ ^]^ In addition, Nav1.6 has also been reported to be involved in pain signaling and inhibition of Nav1.6 holds potential analgesic effects.^[^ ^36^ ^]^ We hypothesize that sparing Nav1.6 may enhance the therapeutic profile of mGpTx1‐SA, whereas on‐target toxicity (mediated by Nav1.6) may occur when receptor occupancy reaches excessively high levels. Increasingly, studies suggest that a network of multiple ion channels regulates pain signaling, and targeting a single channel may not always be sufficient for effective pain management.^[^ ^5^, ^12^, ^37^ ^]^ Therefore, targeting multiple channels may be an alternative to achieve effective treatment of pain, while balancing efficacy and safety.

Although mGpTx1‐SA has demonstrated enhanced safety and analgesic efficacy, there are still certain limitations in this study. (1) These findings are primarily based on results from ion channel levels derived from different species and rodent models. However, rodents and humans exhibit significant differences in physiology, metabolism, and genetic makeup, which can profoundly affect the behavior and efficacy of therapeutic agents. (2) The selectivity of mGpTx1‐SA toward Nav1.6 remains limited, and there is still a potential risk of Nav1.6‐related side effects when used at high doses, which we did not test.

## 4 Experimental Section

### Peptide Synthesis, Oxidative Folding, and Purification

Cl6b, Ca2a, HNTX‐IV, and HNTX‐VIIa were isolated from the venom of spider *Cyriopagopus longipes, Cyriopagopus albostriatus* and *Ornithoctonus hainana*, respectively. HWTX‐IV and ProTx‐II were purchased from AbMole BioScience. For HNTX‐VIIa, the amino acid sequences were obtained by MALDI–TOF MS/MS analysis, and searching the MS/MS data in the Swiss–Prot database matched HNTX‐VIIa (ID: H7A01_CYRHA). As for HNTX‐III, HNTX‐VIIa, mGpTx1, mGpTx1‐R25A, mGpTx1‐SA, and HNTX‐III analogues, they were synthesized manually using a Fmoc [N‐(9‐fluorenyl)methoxycarbonyl]/tert‐butyl strategy and HOBt/TBTU/NMM coupling method. Synthetic crude products were purified by semipreparative reverse‐phase high‐performance liquid chromatography (RP‐HPLC) using an Ultimate XB‐C18 column (300 Å, 10 mm × 250 mm, Welch Materials Inc., Shanghai, China) on the Hanbon HPLC system (Hanbon Sci&Tech., Jiangsu, China). A linear gradient of solvent A (0.1% trifluoroacetic acid (TFA) in acetonitrile) in solvent B (0.1% TFA acid in water) was employed at a flow rate of 3 mL min^−1^: starting with 15% A for 5 min, followed by a gradient increase to 45% A over 30 min. Absorbance was monitored at 215 nm. Desired fractions containing target peptides were concentrated and lyophilized before folding. Target peptides were dissolved in ddH_2_O and diluted in freshly prepared oxidative folding buffer consisting of 0.1 M NaCl, 0.1 M Tris‐HCl (pH 8.0), 5 mM GSH, and 0.5 mM GSSG (pH 7.5 with HCl). After incubating the solution at 4 °C for 24 h, the reaction was terminated by adding TFA to a final concentration of 0.1%. The desired oxidized peptide was then isolated by RP‐HPLC purification as described above. The molecular weights of the target peptides or oxidized peptides were determined using matrix‐assisted laser desorption/ionization‐time‐of‐flight mass spectrometry (MALDI–TOF‐TOF MS) (AB SCIEX TOF/TOF 5800 system, Applied Biosystems, USA) (Data see Supporting Information).

### Plasmid Constructs and Mutagenesis

Nav channels in this study were kindly gifts from Dr. Theodore R. Cummins (Stark Neurosciences Research Institute, Indiana University School of Medicine, United States). The following potassium (Kv) and calcium (Cav) channels were subcloned into the vector pCDNA 3.1 or pCMV‐blank: hKv1.1, hKv1.3, rKv1.4, hKv1.5, rKv2.1, hKv3.1, hKv3.4, mKv4.1, rKv4.2, rKv4.3, hKv7.2, hCav2.1, and hCav3.1‐3.3. Mutants of rKv4.2 were generated via site‐directed mutagenesis. In brief, primers containing the mutation site at the 5′ end were used to amplify rKv4.2 using the KOD FX PCR kit (Toyobo Co, Dalian, China). The resulting product was then digested by DpnI (Thermo Fisher Scientific) to degrade the template DNA. Subsequently, 10 µL of the digestion mix was directly used to transform 100 µL DH5α chemical competent cells. Correctly resistant colonies were screened and DNA sequencing was performed to confirm the mutations.

### Cell Culture and Transfection

ND7/23 and HEK293T cells were cultured in a 5% CO_2_ incubator at 37 °C and maintained with Dulbecco's modified Eagle's medium (DMEM, GIBCO) supplemented with 10% fetal bovine serum (GIBCO), 1% penicillin (GIBCO) and 1% strep‐tomycin (GIBCO). CHO‐K1 cells were maintained in DMEM‐F12 (GIBCO) mixed medium (1:1) with 10% FBS and cultured under the same conditions. Transient transfection of channel plasmids was performed using Lipofectamine 2000 (Thermo Fisher Scientific) according to the manufacturer's instructions. hNav1.2, rNav1.3, hNav1.4 and hNav1.5 channel plasmids were cotransfected with eGFP into HEK293T cells. hNav1.7 together with β1 and β2‐eGFP plasmids were transfected in HEK293T cells. But, mNav1.6 mutant with resistance to tetrodotoxin (Y362S, TTX‐R)^[^ ^25^ ^]^ and rNav1.8 were transfected in ND7/23 with eGFP. For hKv1.3, rKv1.4, hKv1.5, rKv2.1, hKv3.1, hKv3.4, mKv4.1, rKv4.3, hCav3.1‐3.3, hCav2.1, rKv4.2, and rKv4.3 mutants were cotransfected with eGFP into HEK293T cells. hKv1.1 and hKv7.2 together with eGFP were transfected in CHO‐K1 cells.

### Electrophysiology

Whole‐cell patch‐clamp recordings were conducted using an EPC‐10 USB patch‐clamp amplifier operated by PatchMaster software (HEKA Elektronik, Lambrecht, Germany). Fire‐polished electrodes (2.0–2.5 MΩ) were prepared in a PC‐10 puller (NARISHIGE, Tokyo, Japan) using a two‐step program. Capacity transients were canceled, and series resistance was maintained below 10 MΩ with voltage errors minimized by 80% series resistance compensation in the whole‐cell configuration. Recordings were performed at room temperature (25 ± 2 °C). For recording Kv channels in HEK293T and CHOK1 cells, the bath solution contains (in mM): 140 NaCl, 5 KCl, 2 CaCl_2_, 1 MgCl_2_, 10 HEPES and 10 D‐glucose (pH 7.4 with NaOH), while the pipette solution contained (in mM): 140 KCl, 2.5 MgCl_2_, 10 HEPES and 10 EGTA (pH 7.4 with KOH). For recording Nav channels in HEK293T and ND7/23 cells, the bath solution contains (in mM): 140 NaCl, 5 KCl, 2 CaCl_2_, 1 MgCl_2_, 10 HEPES and 10 D‐glucose (pH 7.4 with NaOH), and the corresponding pipette solution contains (in mM): 140 CsF, 1 EGTA, 10 NaCl and 10 HEPES (pH 7.4 with CsOH). Additionally, for recording the mNav1.6 (Y362S, TTX‐R) or rNav1.8 current that is expressed on ND7/23 cells, the extracellular solution was supplemented with 1 µM TTX to block endogenous TTX‐S Na^+^ currents in ND7/23 cells. For recording Cav channels in HEK293T cells, the bath solution contains (in mM): 100 NaCl, 20 TEA‐Cl, 5 Ba_2_Cl, 1 MgCl_2_, 10 HEPES, and 10 D‐glucose (pH 7.4 with NaOH), while the pipette solution contains (in mM):120 CsMeSO_4_, 10 HEPES, 5 Mg‐ATP, and 11 EGTA (pH 7.2 with CsOH). For cardiomyocyte action potential recording, the bath solution contains (in mM): 140 NaCl, 5 KCl, 1 MgCl_2_·6H_2_O, 1.8 CaCl_2_·2H_2_O, 5 HEPES, 10 Dextrose (pH 7.4 with NaOH), and the corresponding pipette solution contains (in mM): 110 Aspartic‐Acid, 20 KCl, 5 Na_2_‐Phosphocreatine, 10 HEPES, 5 EGTA, 5 Mg‐ATP, 0.1 GTP (pH 7.2 with KOH). For recording TTX‐S Nav currents of DRG neurons, the bath solution contains (in mM): 30 NaCl, 1 MgCl_2_, 1.8 CaCl_2_, 5 CsCl, 5 KCl, 25 D‐glucose, 5 HEPES, 0.1 CdCl_2_ and TEA‐Cl (pH 7.3 with NaOH), and the pipette solution contains (in mM): 135 CsCl, 10 NaCl and 5 HEPES (pH 7.3 with CsOH). For recording the TTX‐R Nav currents in DRG neurons, the extracellular solution was supplemented with 1 µM TTX to block endogenous TTX‐S Na^+^ currents. For recording the voltage‐gated K^+^ currents in DRG neurons, the bath solution contained (in mM): 130 choline chloride, 5 KCl, 2 MgCl_2_, 2 CaCl_2_, 10 HEPES, 10 D‐glucose, (pH 7.3 with Tris), and the pipette solution contained (in mM): 120 KCl, 20 NMG, 10 EGTA, 10 HEPES (pH 7.3 with KOH). For current‐clamp recording, the extracellular solution contained (in mM): 140 NaCl, 3 KCl, 2 CaCl_2_, 2 MgCl_2_, and 10 HEPES (pH 7.3 with NaOH); the pipette solution contained (in mM): 140 KCl, 0.5 EGTA, 5 HEPES and 2 Mg‐ATP (pH 7.3 with KOH). For recording Nav channels currents of primary cardiomyocytes, the bath solution contained (in mM): 30 NaCl, 1 MgCl_2_, 1.8 CaCl_2_, 110 CsCl, 10 D‐glucose, and 10 HEPES (pH 7.3 with CsOH), and the pipette solution contains (in mM): 130 CsCl, 10 NaCl, 5 HEPES and 1 MgCl_2_ (pH 7.2 with CsOH). For recording the Kv currents in primary cardiomyocytes, the bath solution contained (in mM): 140 NaCl, 5 KCl, 2 CaCl_2_, 1 MgCl_2_, 10 HEPES and 10 D‐glucose, 0.0002 CdCl_2_ and 0.0003 BaCl_2_ (pH 7.4 with NaOH), and the pipette solution contained (in mM) 140 KCl, 2.5 MgCl_2_, 10 HEPES and 10 EGTA (pH 7.4 with KOH). All reagents were obtained from Sigma.

For recording the hKv1.1, hKv1.3, rKv1.4, hKv1.5, rKv2.1, hKv3.1, hKv3.4, rKv4.2, rKv4.3, mKv4.1 and hKv7.2 channel's currents, cells were held at ‐80 mV and stepped to potential of 0 mV for 300‐ms every 5‐s.

Cells were held at ‐90 mV, and hNav1.2, rNav1.3, hNav1.4, hNav1.5, mNav1.6 (Y362S, TTX‐R), hNav1.7 currents were elicited by the potential of 0 mV for 50‐ms, and rNav1.8 currents were elicited by the potential of 20 mV for 50‐ms. Each sweep has a 5‐s interval. For hCav2.1 and hCav3.1‐3.3, cells were held at ‐120 mV, and the currents were elicited by potential of 0 mV for 200‐ms.

To generate rKv4.2 and rKv4.3 activation curves, cells were held at ‐80 mV and stepped to potentials of −80 to +100 mV in 10 mV increments for 300‐ms every 5‐s. The conductance‐voltage (G‐V) curves were obtained by calculating the conductance (G) at each voltage (V) using the equation: G = I/(V‐V_rev_). The reversal potential (V_rev_) for K^+^ current was determined to be ‐85 mV using the Nernst equation. G‐V curves were fitted with a Boltzmann equation using Prism 9.5 (Version 9.5.0, GraphPad Software).

To measure steady‐state inactivation of rKv4.2 and rKv4.3 channels, the cell was held at −80 mV, and a series of pre‐pulses (−80 to +100 mV in 10 mV increments for 1000‐ms) were applied. This was followed by +60 mV test pulse for 300‐ms to assess the available non‐inactivated currents and the sweep interval was set to 5 s. Currents were normalized (I/I_max_) and fitted with a Boltzmann equation using Prism 9.5 (Version 9.5.0, GraphPad Software). To record cardiomyocyte action potentials, the mode was switched to current clamp and the cell was clamped at 0 pA. Stimulations ranging from 0 to 3000 pA were applied for 2–5 ms with a 1 s sweep (1 Hz). To record DRG neurons APs, the mode was switched to current clamp and the cell was clamped at 0 pA. Stimulations ranging from 0 pA to 120 pA were applied for 1 s with a 5 s sweep (1 Hz).

All concentration–response curves were fitted by a Hill logistic equation to estimate the potency (IC_50_) of the toxin.

### Animal Experiments

*Experimental Animals and Study Approval*: Healthy C57BL/6 mice (weight 18–20 g, 6–8 weeks), ICR mice (weight 18–20 g, 6–8 weeks), and Sprague‐Dawley (SD) rats (weight 220–250 g, 6–8 weeks) were purchased at the Experimental Animal Center of SLac‐kinda. For C57BL/6 and ICR, both female and male mice were used in combination. These mice were housed under controlled conditions with a constant temperature of 24 °C and a 12‐hour light/dark cycle. They were provided with standard laboratory food and water ad libitum. All dosing and scoring activities were carried out by experimenters who were blinded to the treatment conditions.

All of the animal experiments were performed in accordance with the Guidelines for Laboratory Animal Research set by Hunan Normal University. The experiments were approved by the Institutional Animal Care and Use Committee of the College of Medicine, Hunan Normal University (identification code: 2 021 023; date of approval: 9 March 2021).

*Evaluation of Analgesic Activity*: DRG neurons isolation and culture: C57BL/6 mice (weight 18–20 g, 6–8 weeks, both sex) were euthanized via cervical dislocation under anesthesia. Then, DRG neurons were collected from the lumbar spinal cord L4–L5 and dissociated by enzymatic treatment with collagenase (0.32 mg mL^−1^) and trypsin (0.15 mg mL^−1^) at 37 °C for 30 min. After centrifuging at 800 rpm for 5 min at room temperature, Suspension cells were seeded onto poly‐L‐lysine coated coverslips and cultured in DMEM (Gibco) containing 10% fetal bovine serum (Gibco) and at 37 °C in a humidified incubator with 5% CO_2_ for 3 h before whole‐cell patch‐clamp recording.

In the Complete Freund's adjuvant (CFA)–induced inflammatory pain modelinflammation is induced by injecting 15 µL of CFA into the intraplantar surface of the right hind paw of C57BL/6 mice (weight 18–20 g, 6–8 weeks, both sex).^[^ ^38^ ^]^ Mechanical paw withdrawal thresholds are then measured using manual von Frey (Stoelting Co) after 2 days of CFA injection. Subsequently, mGpTx1: 40 µg kg^−1^ (4 males and 4 females) and 60 µg kg^−1^ (5 males and 4 females), mGpTx1‐SA: 40 µg kg^−1^ (5 males and 5 females) and 60 µg kg^−1^ (5 males and 4 females), morphine (3 mg kg^−1^ (4 males and 4 females), or saline (3 males and 3 females) are administered on day 2 after CFA injection.

In the spared nerve injury (SNI) neuropathic pain model,^[^ ^39^ ^]^ C57BL/6 mice (weight 18–20 g, 6–8 weeks, both sex) were anesthetized with 3% halothane. The thigh was incised parallel to the femur, and the muscles were bluntly separated to expose the sciatic nerve. The sciatic nerve is ligated with 6.0 silk sutures. Fourteen days after establishing the SNI model, mGpTx1 (60 µg kg^−1^, 4 males and 3 females), mGpTx1‐SA (60 µg kg^−1^, 4 males and 4 females), morphine (5 mg kg^−1^, 4 males and 4 females), or saline (2 males and 1 females) is administered. The paw withdrawal threshold to mechanical stimuli is measured between 0 and 180 min after drug administration.

*Cardiac Toxicity Assay*: *Atrial Myocytes Isolation*: Isolation of cardiomyocytes from 6 SD rats (weight 220–250 g, 6–8 weeks, male) was performed with some modifications as previously reported.^[^ ^40^ ^]^ Briefly, SD rats were injected with heparin sodium (3125 U/kg i.p.) and anesthetized after 15–20 min. The hearts were then excised and mounted on a Langendorff perfusion apparatus. Remaining blood in the hearts was washed away with Ca^2+^‐free Tyrode's solution (in mM: 140 NaCl, 5 KCl, 1 MgCl_2_, 5 HEPES, 10 Glucose) via the aorta for 3–5 min at a flow rate of 10 mL min^−1^. The perfusion solution was then switched to the digestion solution (40 mL Ca^2+^‐free Tyrode's, 40 mg BSA, and 18 mg type II collagenase), and perfused for 18–20 min until the heart became soft. Following this, the heart was then perfused with KB solution (in mM: 120 K‐Glutamic acid, 10 KCl, 10 KH_2_PO_4_, 1.8 MgSO_4_·7H_2_O, 0.5 EGTA, 20 D‐Glucose, 10 Taurine, 10 HEPES, pH 7.2 with KOH and osmolality ± 300 mOsm L^−1^ with mannitol) to wash out the digestion solution. The heart was then cut into pieces, passed through a 200‐mesh sieve, and the isolated cardiomyocytes were collected by centrifugation for subsequent experiments.

*Surface Electrocardiographic (ECG) Recording*: ICR mice (weight 18–20 g, 6–8 weeks, both sex) were lightly anesthetized with 3% halothane for Surface electrocardiograms (ECG), which were recorded by placing dry electrodes carefully wrapped around each of the mouse's four limbs. The data of physiological effects of intravenous administration of 0.3 mg kg^−1^ mGpTx1 (4 males and 4 females) or 2 mg kg^−1^ mGpTx1‐SA (3 males and 2 females) for 5 min were collected by PowerLab 2/28 and LabChart 8 (AD Instruments, Bella Vista, Australia) and analyzed standard ECG parameters by along with its accompanying ECG analysis plug‐in. For each mouse, the data of 8 cardiac cycles before and after administration were averaged for analysis.

*In Vivo Safety Evaluation*: *Rotarod Test*: ICR mice (weight 18–20 g, 6–8 weeks, both sex) were trained on a rotarod at a constant speed of 20 rpm for 8 min per day. After 4 days of training, mice whose motor performance deviated significantly from the mean value were excluded from further experiments. On the 5th day, the remaining mice were randomly divided into two groups: Saline (3 males and 2 females) and 2 mg kg^−1^ mGpTx1‐SA (3 males and 3 females). All groups were administered intraperitoneally and allowed 20 minutes for the drug to take effect before behavioral tests were conducted. The total motor time of the animals during 300 s was recorded for analysis.

*Forced Swimming Test*: ICR mice (weight 18–20 g, 6–8 weeks, both sex) were subjected to forced swimming training for 8 min per day over a span of 4 days. Mice whose swimming time significantly deviated from the mean value during training were excluded from further experiments. On the 5th day, the remaining mice were randomly assigned to one of two groups: Saline (3 males and 2 females) and the 2 mg kg^−1^ mGpTx1‐SA (3 males and 2 females). All groups received intraperitoneal administration and were allowed 20 min for the drug to take effect before behavioral tests commenced. Swimming time was recorded for a duration of 4 min within a 6‐minute timeframe following drug administration.

*Open Field Test*: The C57BL/6 mice (weight 18–20 g, 6–8 weeks, both sex) were randomly assigned to one of two groups: Saline (2 males and 2 females) and 2 mg kg^−1^ mGpTx1‐SA (2 males and 2 females). All mice received intraperitoneal administration of the respective substances and were allowed 20 min for the drugs to take effect. Following drug administration, the mice were individually placed in square transparent chambers (50 × 50 cm). They were allowed to explore freely under normal light. The movements and trajectories of the mice were recorded using video and computer systems over a 2‐minute period for subsequent analysis.

*Small Intestinal Propulsion Test*: ICR mice (weight 18–20 g, 6–8 weeks, both sex) were subjected to a 24‐hour fasting period to ensure gastrointestinal emptying. Subsequently, the mice were randomly assigned to one of two groups: Saline (2 males and 2 females) and 2 mg kg^−1^ mGpTx1‐SA (2 males and 2 females). All groups received intraperitoneal administration of their respective substances and were allowed 20 minutes for the drugs to take effect. Following drug administration, each mouse received a pre‐prepared nutritive semi‐solid paste (10 g of sodium carboxymethylcellulose, 16 g of milk powder, 8 g of sucrose, 8 g of starch, and 5 g of activated charcoal, stirred well in 250 mL of ddH_2_O) via gavage (0.5 mL per 20 g). After 20 min, the mice were euthanized, and their small intestines were carefully removed. The distance from the pylorus to the ileum was measured along the full length of the small intestine. Additionally, the distance from the pylorus to the front of the black semi‐solid paste was measured. Small intestinal propulsion rate (%) = length of carbon paste propulsion (cm) × 100% / total length of small intestine (cm).

### Data Analysis

Data were analyzed with Mega (Version 6.0), Pymol (Version 2.40), PatchMaster v2×73 (HEKA Elektronik), IgorPro6 (Version 6.1.0.9, WaveMetrics, Lake Oswego, OR, USA), Prism 9.5 (Version 9.5.0, GraphPad Software) and Office Excel 2010 (Version 14.0.4760.1000, Microsoft, USA). All values are shown as mean ± S.E.M., and n represents the number of animals or cells examined. Two‐tailed t‐test, One‐way ANOVA, and two‐way ANOVA were used to assess the difference between multiple groups or two groups. In figure legends, the statistical method to a specific experiment is mentioned, and *p* values are also shown. Statistical analyses were performed with Prism 9.5 software.

## Conflict of Interest

The authors declare no conflict of interest.

## Author Contributions

S.L., X.Z., M.W., and G.W. contributed equally to this work. X.Z. and Z.L. performed conceptualization. X.Z. and Z.L. performed data curation. X.Z., S.L., and Y.Z. performed formal analysis. X.Z., Z.L., Y.Z., and S.L. performed funding acquisition. S.L., X.Z., M.W., G.W., L.W., X.F., H.W., R.L., M.L., J.J., W.W., L.Y., X.L., D.P., L.Y., Q.Z., M.C., X.D., G.H., Y.Z. performed investigation. X.Z. performed methodology. X.Z., Z.L., Y.Z. performed supervision. X.Z., S.L. performed writing – original draft. X.Z. and Z.L. performed writing – review & editing.

## Supporting information



Supporting Information

## Data Availability

The data that support the findings of this study are available from the corresponding author upon reasonable request.
