# Supplementary material for: Optimizing Nav1.7‐Targeted Analgesics: Revealing Off‐Target Effects of Spider Venom‐Derived Peptide Toxins and Engineering Strategies for Improvement
Source: Adv Sci (Weinh). 2024 Sep 9;11(42):2406656. doi: 10.1002/advs.202406656 (PMC11558128; doi:10.1002/advs.202406656)
Supplement: Supplementary file 1 — Supporting Information [file ADVS-11-2406656-s001.docx]

**Supplementary Figures and Figure legends**

**
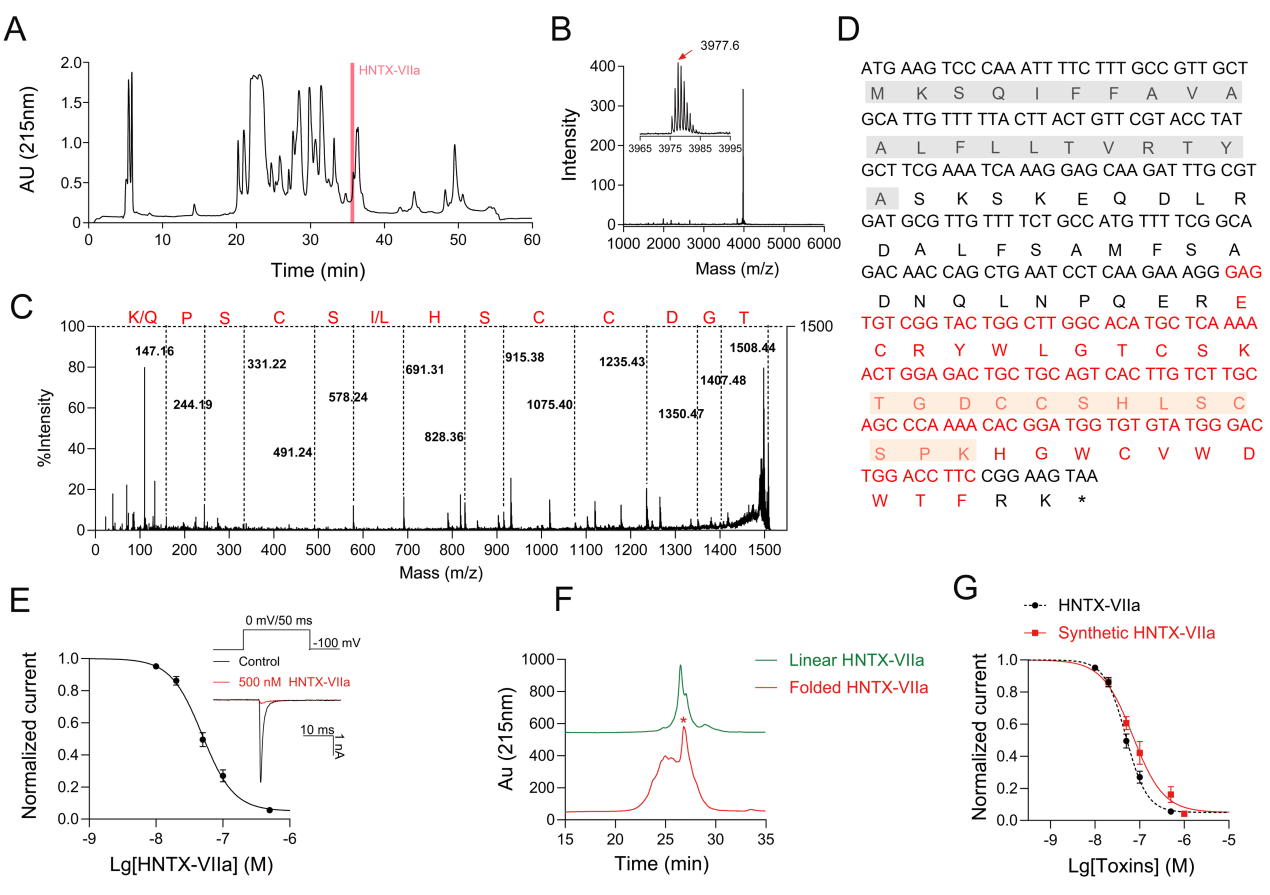
**

**Figure S1.** The isolation and activity assessment of HNTX-VIIa. (**A**) RP-HPLC profile of the venom from the spider *Ornithoctonus hainana*. The fraction of HNTX-VIIa is highlighted in pink. (**B**) The molecular weight of HNTX-VIIa is 3977.6 Da (M+H^+^), determined by MALDI–TOF MS. (**C**) HNTX-VIIa was reduced, alkylated and digested with trypsin, shown is a representative MALDI–TOF MS/MS analysis of the parental ion with the m/z of 1508.44, and searching the MS/MS data in the Swiss–Prot database matched HNTX-VIIa (ID: H7A01_CYRHA). (**D**) cDNA and protein sequence of HNTX-VIIa, marked in gray is the signal peptide, partial sequence of HNTX-VIIa (TGDCCSHLSCSPK) was obtained by MALDI–TOF MS/MS analysis and marked in orange, mature sequence of HNTX-VIIa shown in red and its C-terminal residue Phe34 was amidated. (**E**) Concentration-dependent inhibitory curves show the effect of native HNTX-VIIa on hNav1.7 (n = 5). *Inset* shows representative current traces in the presence (red) or absence (black) of 500 nM HNTX-VIIa. (**F**) RP-HPLC chromatograms of the linear peptide HNTX-VIIa (top, green) and the refolded one (bottom, red). The target fraction is marked with *. (**G**) Concentration -dependent inhibitory curves show the effect of the synthetic HNTX-VIIa on hNav1.7 (red, n = 3). Data are presented as the mean ± S.E.M.


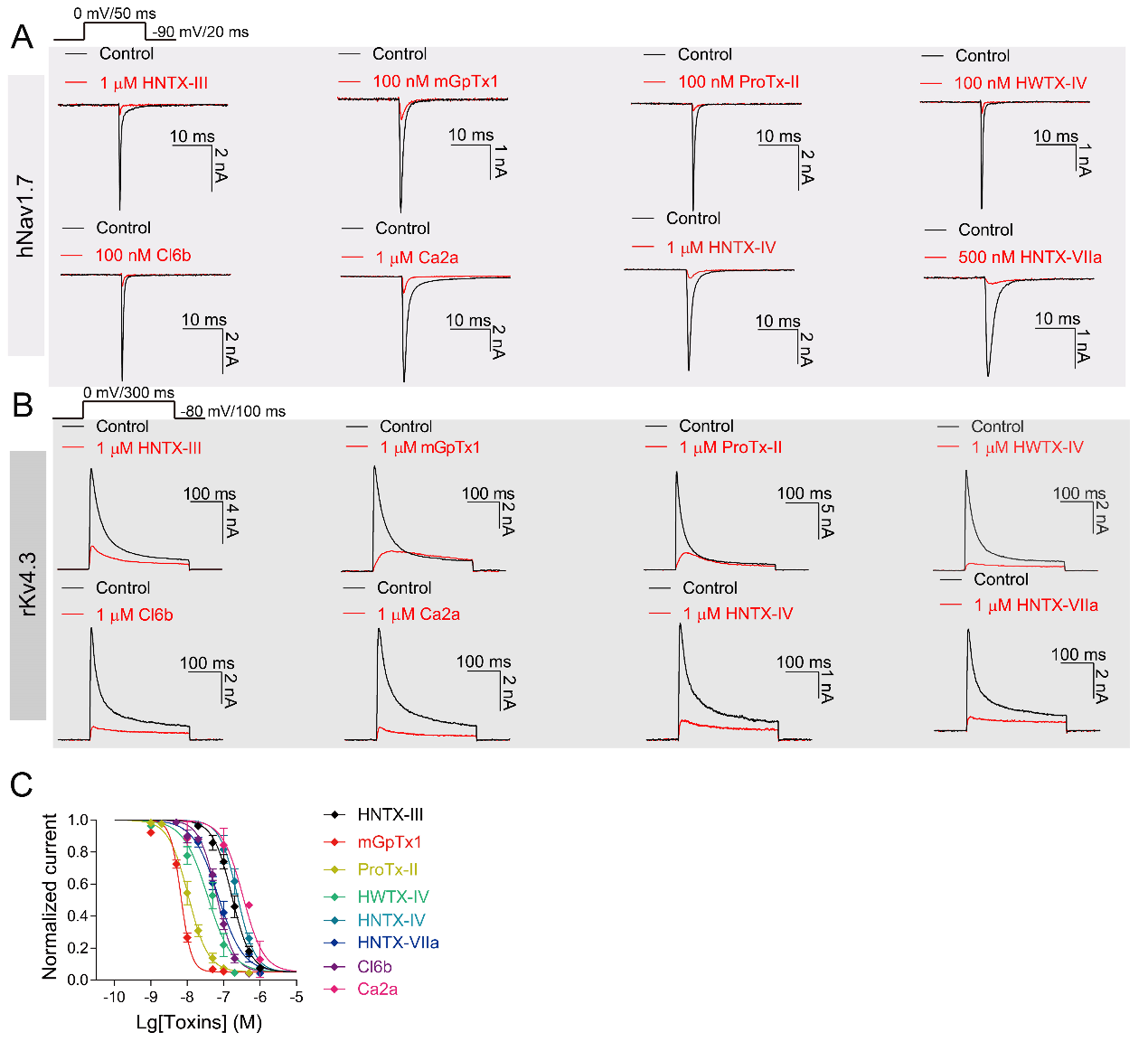


**Figure S2**. The effects of HNTX-III, mGpTx1, ProTx-II, HWTX-IV, Cl6b, Ca2a, HNTX-IV and HNTX-VIIa on rKv4.3 or hNav1.7 currents. (**A**) Representative current traces from HEK293T cells expressing hNav1.7 in the absence (black) and presence (red) of the toxins at the concentrations indicated. The inset shows the currents elicited protocol. (**B**) Representative current traces from HEK293T cells expressing rKv4.3 in the absence (black) and presence (red) of the toxin indicated at the concentration of 1 μM. The inset shows the currents elicited protocol. (**C**) Concentration-dependent inhibitory curves of the eight toxins on hNav1.7 assessed by whole-cell patch-clamp experiments (n = 4-7). Data are represented as mean ± S.E.M.


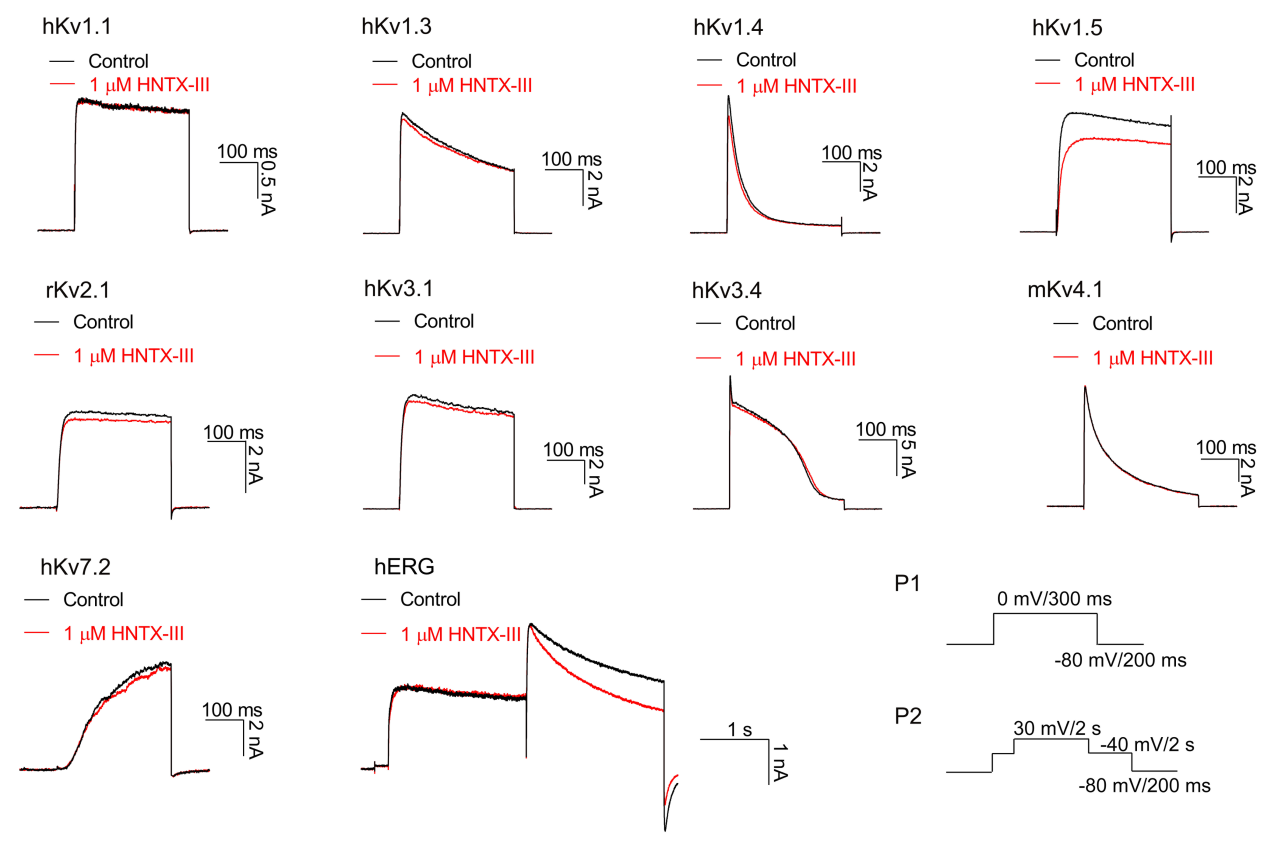


**Figure S3.** The selectivity of HNTX-III on Kv subtypes. Representative current traces from HEK293T cells or CHO cells expressing hKv1.1-1.5, rKv2.1, hKv3.1-3.4, mKv4.1, hKv7.2 and hERG in the absence (black) and presence (red) of 1 μM HNTX-III (n = 3-4). The currents of hKv1.1-1.5, rKv2.1, hKv3.1-3.4, mKv4.1 and hKv7.2 were elicited by protocol P1, and hERG currents were elicited by protocol P2.


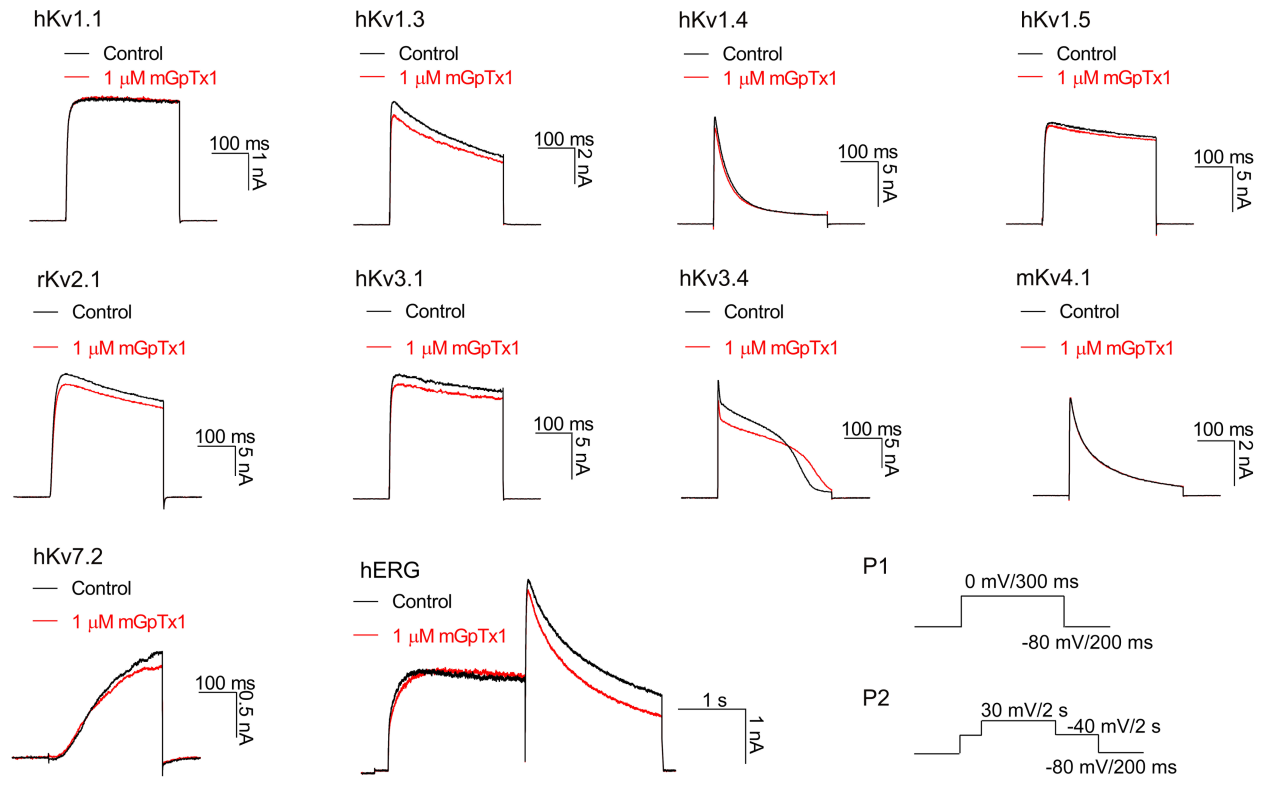


**Figure S4.** The selectivity of mGpTx1 on Kv subtypes. Representative current traces from HEK293T cells or CHO cells expressing hKv1.1-1.5, rKv2.1, hKv3.1-3.4, mKv4.1, hKv7.2 and hERG in the absence (black) and presence (red) of 1 μM mGpTx1 (n = 3-4). The currents of hKv1.1-1.5, rKv2.1, hKv3.1-3.4, mKv4.1 and hKv7.2 were elicited by protocol P1, and hERG currents were elicited by protocol P2.


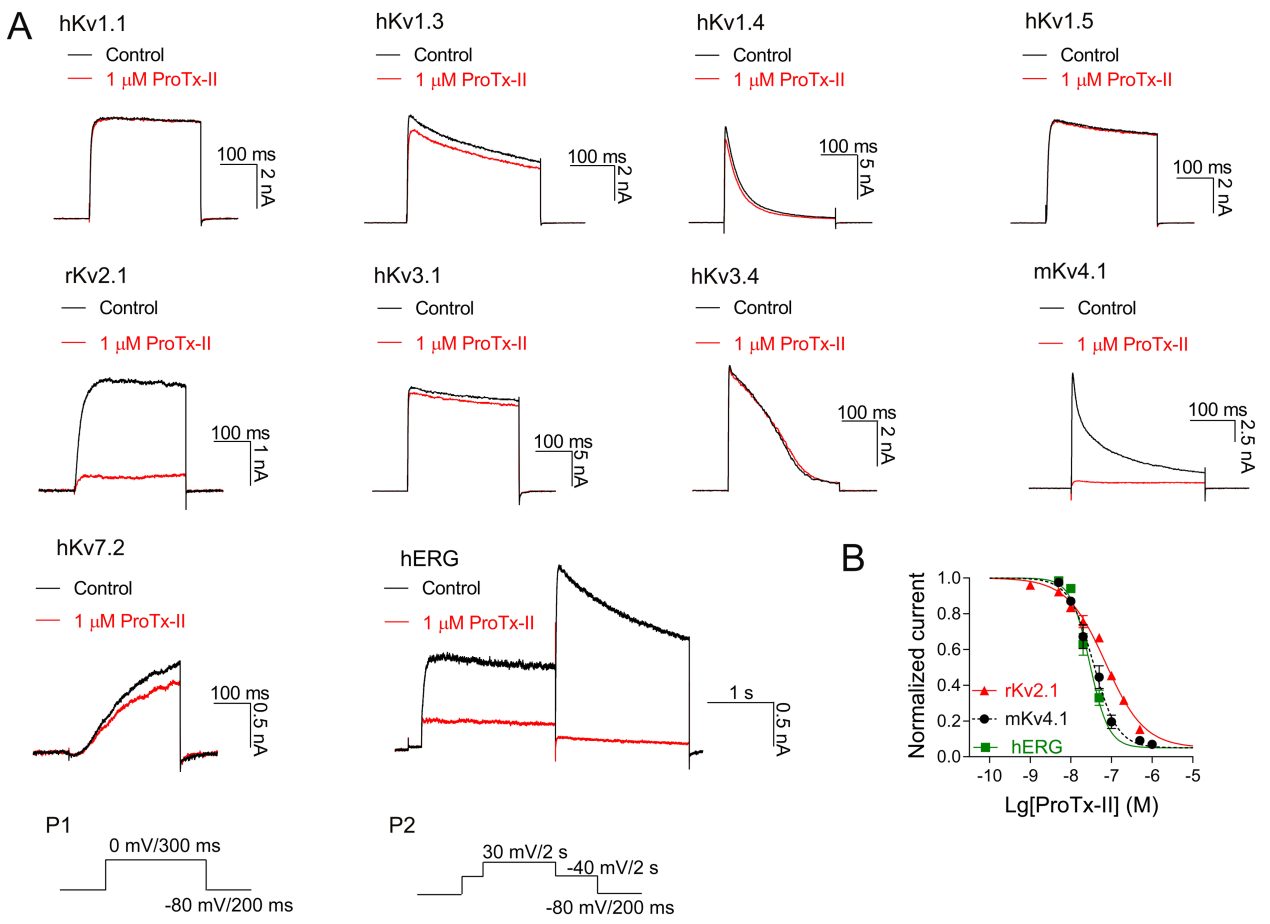


**Figure S5.** The selectivity of ProTx-II on Kv subtypes. (**A**) Representative current traces from HEK293T cells or CHO cells expressing hKv1.1-1.5, rKv2.1, hKv3.1-3.4, mKv4.1, hKv7.2 and hERG in the absence (black) and presence (red) of 1 μM ProTx-II (n = 3-4). The currents of hKv1.1-1.5, rKv2.1, hKv3.1-3.4, mKv4.1 and hKv7.2 were elicited by protocol P1, and hERG currents were elicited by protocol P2. (**B**) Concentration-dependent inhibitory curves show the effects of ProTx-II on mKv4.1 (black, n = 5), rKv2.1 (red, n = 6) and hERG (green, n = 4), respectively. Data are presented as mean ± S.E.M.


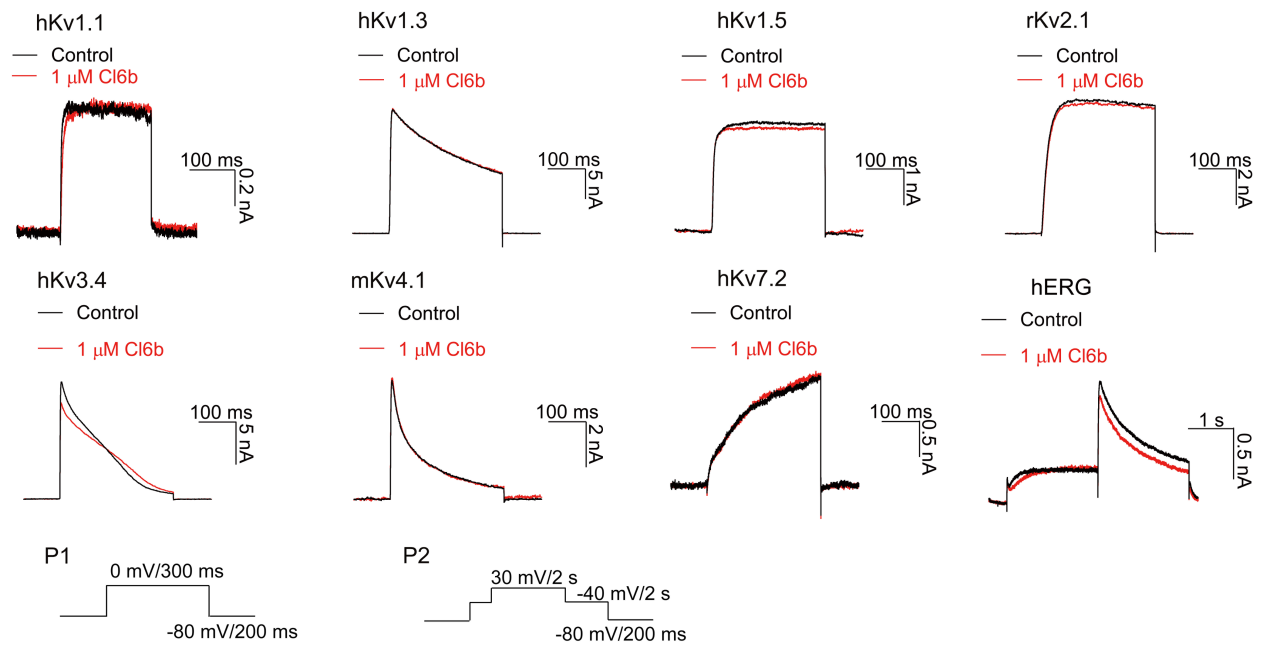


**Figure S6.** The selectivity of Cl6b on Kv subtypes. Representative current traces from HEK293T cells or CHO cells expressing hKv1.1-1.5, rKv2.1, hKv3.4, mKv4.1, hKv7.2 and hERG in the absence (black) and presence (red) of 1 μM Cl6b (n = 3-4). The currents of hKv1.1-1.5, rKv2.1, hKv3.4, mKv4.1 and hKv7.2 were elicited by protocol P1, and hERG currents were elicited by protocol P2.


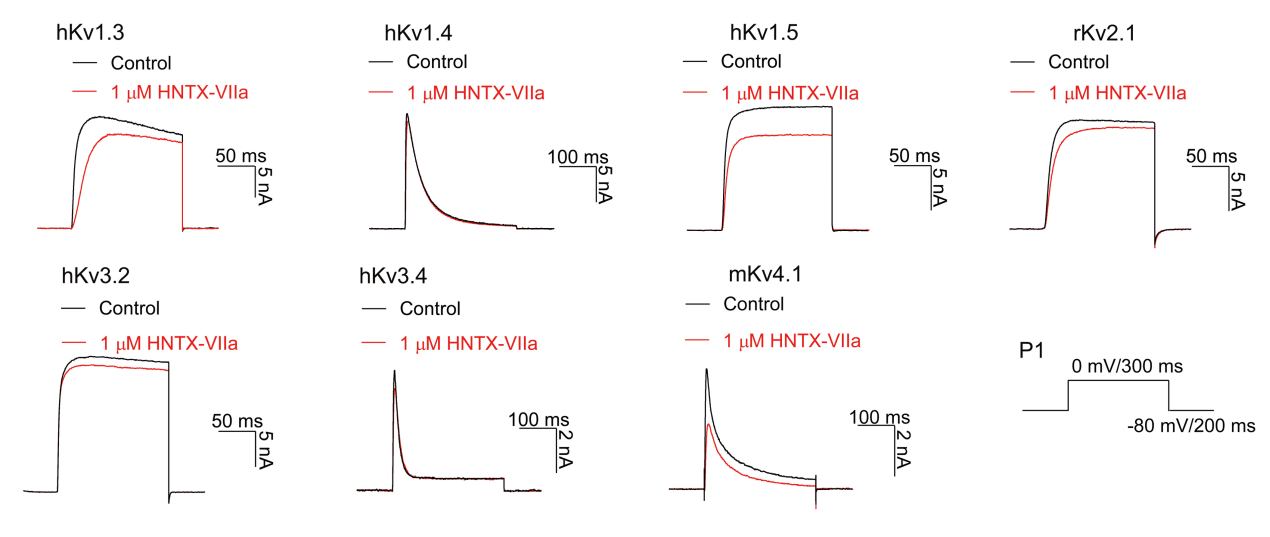


**Figure S7.** The selectivity of HNTX-VIIa on Kv subtypes. Representative current traces from HEK293T cells or CHO cells expressing hKv1.3-1.5, rKv2.1, hKv3.2, hKv3.4 and mKv4.1 in the absence (black) and presence (red) of 1 μM HNTX-VIIa (n = 3-4). The currents of hKv1.3-1.5, rKv2.1, hKv3.2, hKv3.4 and mKv4.1 were elicited by protocol P1.


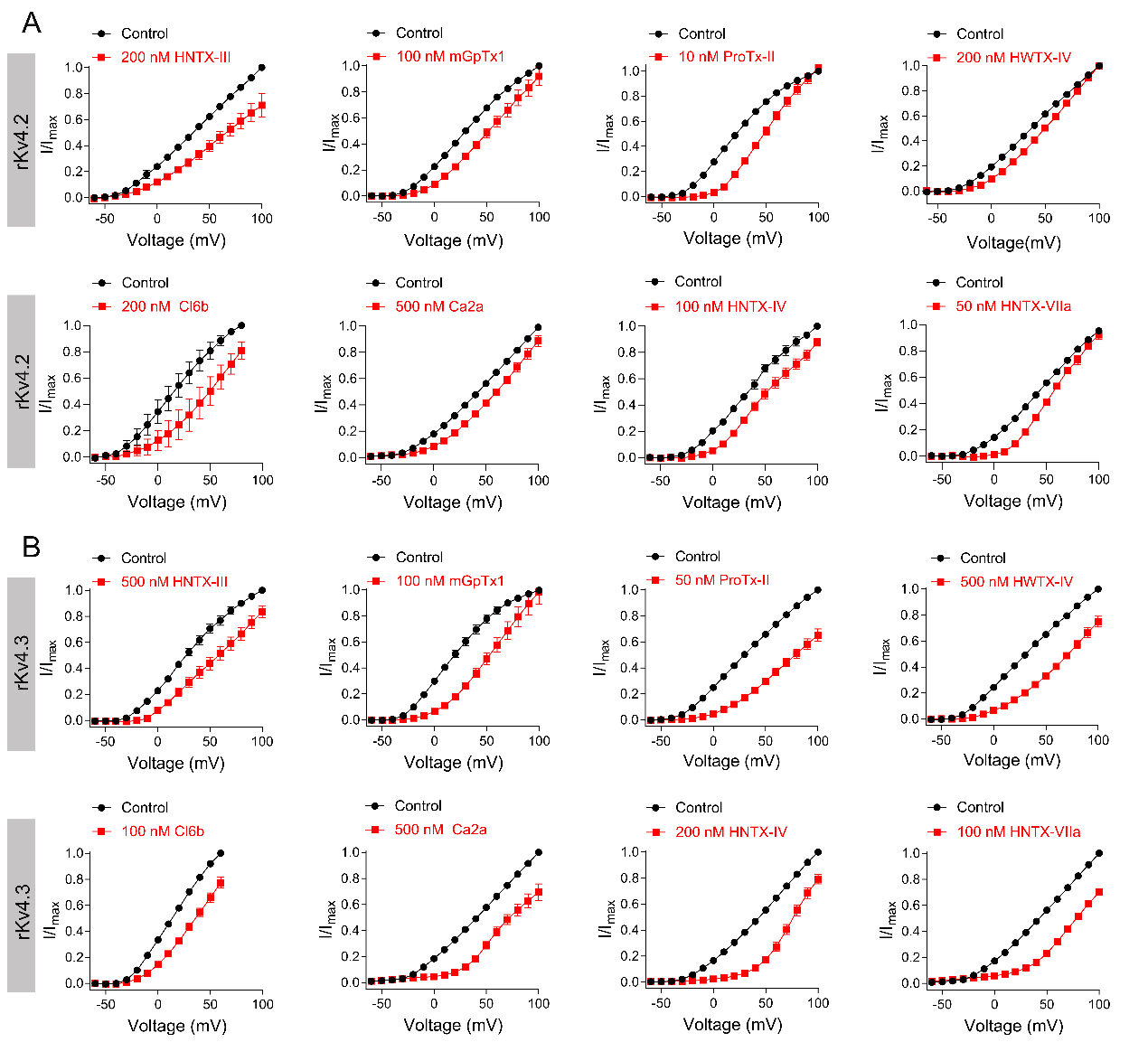


**Figure S8.** The voltage-dependent inhibition of the eight toxins on rKv4.2 or rKv4.3. (**A**) Normalized current-voltage curves of rKv4.2 in the absence (black) and presence (red) of the eight toxins. (n = 4-7). (**B**) Normalized current-voltage curves of rKv4.3 in the absence (black) and presence (red) of the eight toxins. (n = 4-7). Data are presented as mean ± S.E.M.


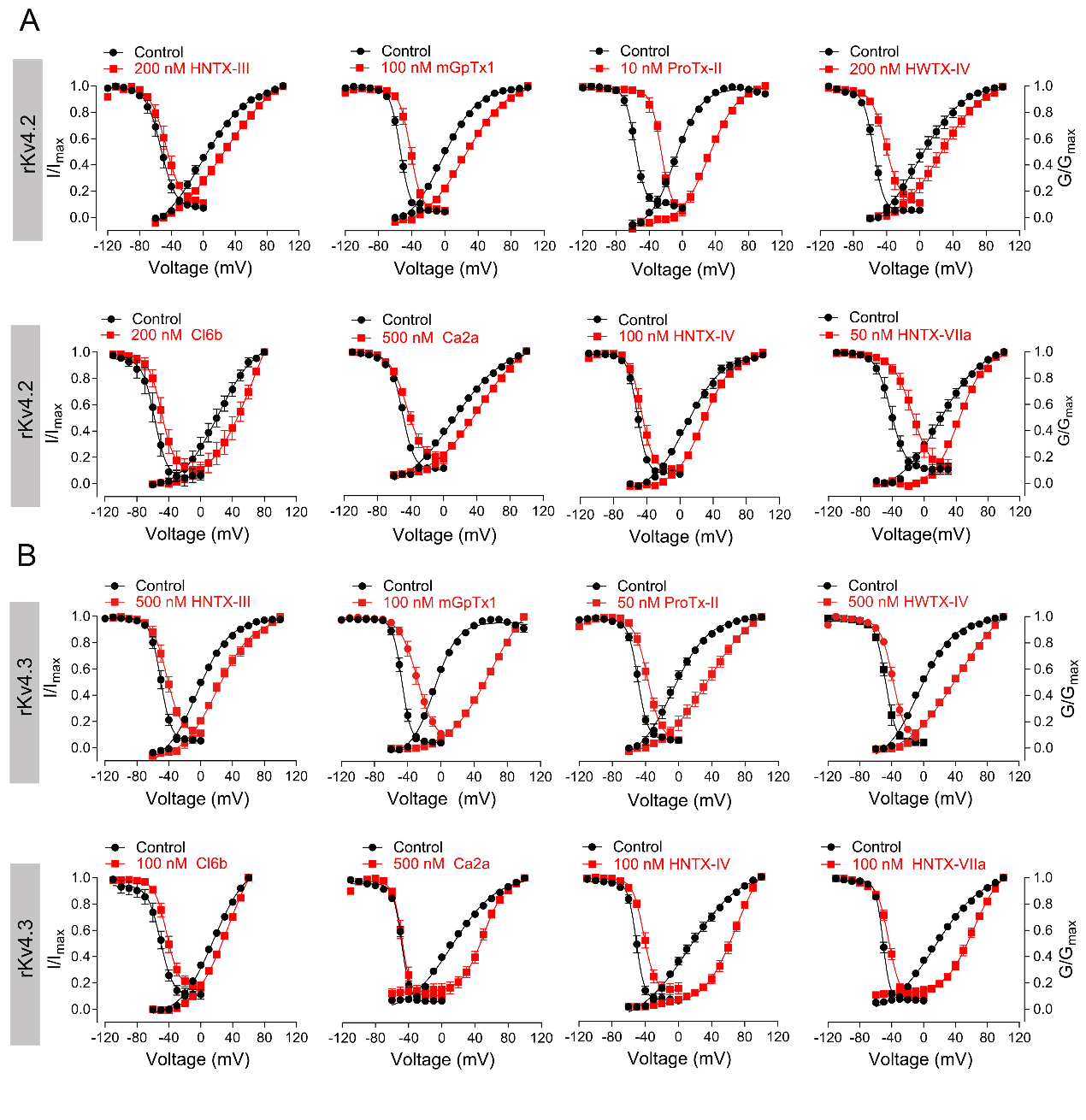


**Figure S9.** The effects of the eight toxins on rKv4.2 or rKv4.3. (**A** and **B**) Voltage-dependent steady-state activation (G/G_max_) and inactivation (I/I_max_) curves of rKv4.2 (**A**) or rKv4.3 (**B**) with the treatment of control (black dots, n = 5-9 for activation, n = 5-9 for inactivation) or the eight toxins (red squares, n = 5-9 for activation, n = 5-9 for inactivation), respectively. Data are represented as mean ± S.E.M.


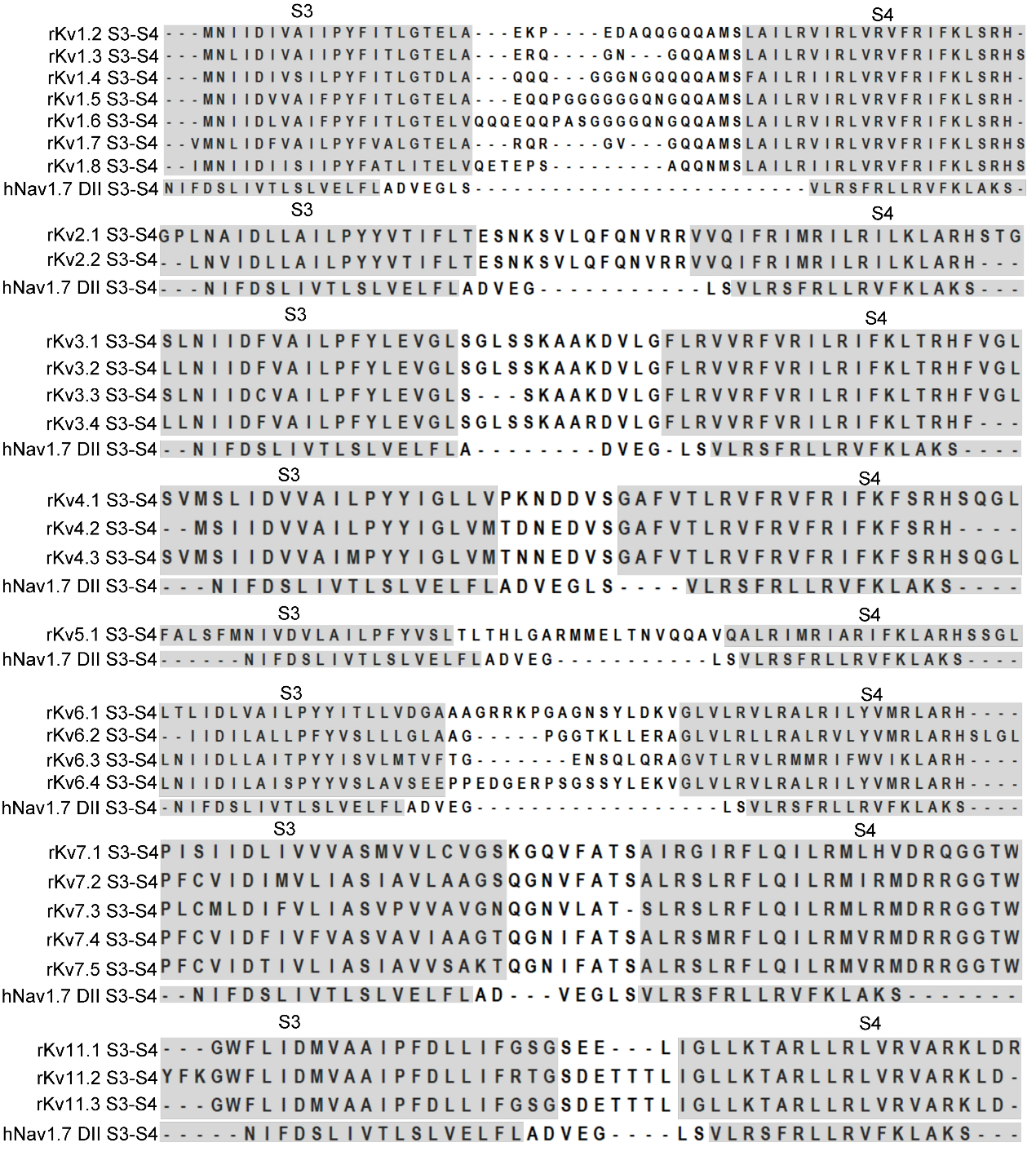


**Figure S10.** Sequence alignment of the S3-S4 regions of hNav1.7 DII and rKv channels from different families by MEGA6.0.


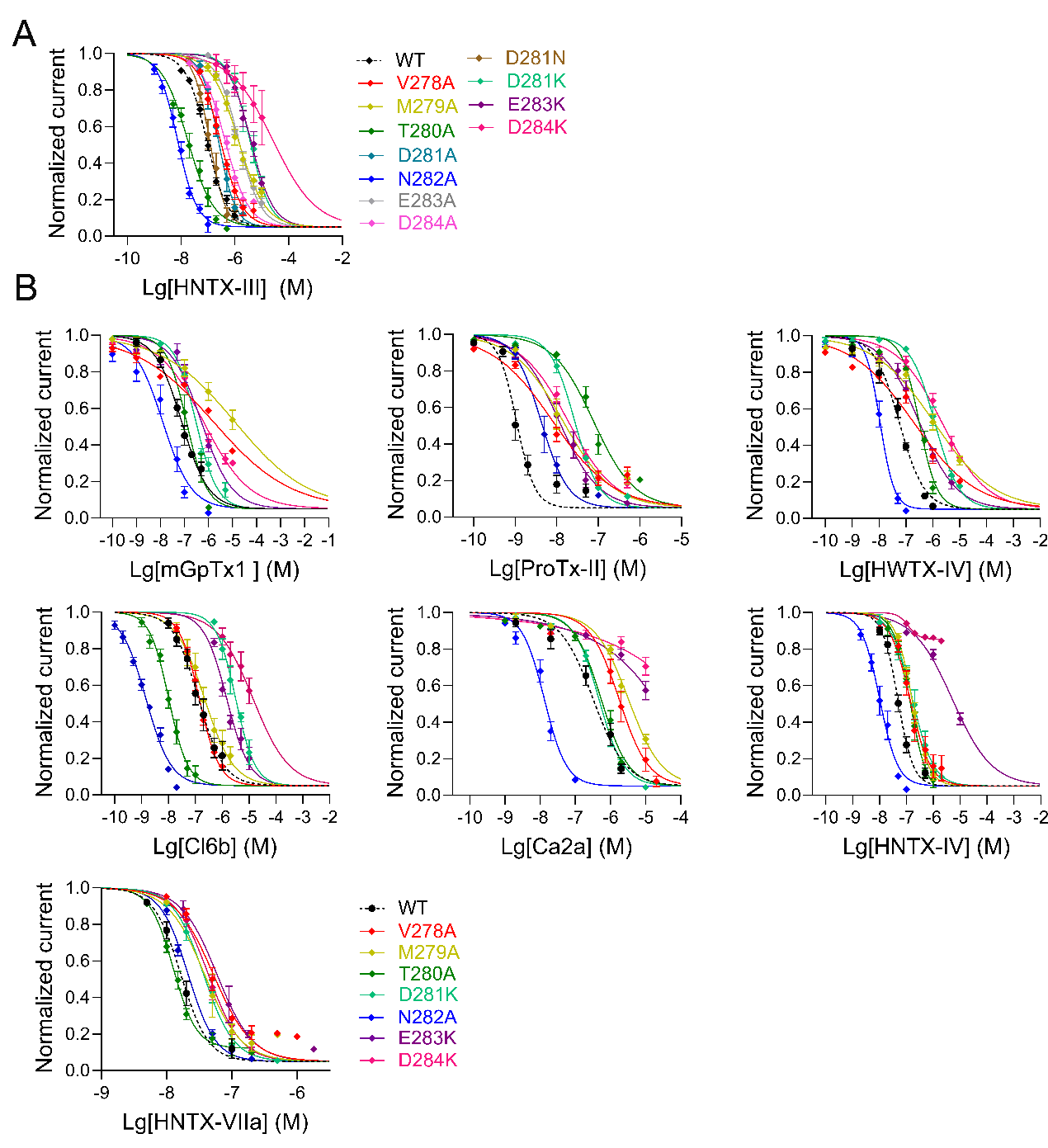


**Figure S11.** The Concentration-dependent inhibitory curves of the eight toxins on rKv4.2 mutants. (**A**) Concentration-dependent inhibitory curves show the effects of HNTX-III on the WT (black) and mutant rKv4.2 channels as labeled (n = 3-7), respectively. (**B**) Concentration-dependent inhibitory curves show the effects of the other seven toxins on the WT and mutant rKv4.2 channels (n = 3-6). Data are presented as mean ± S.E.M.


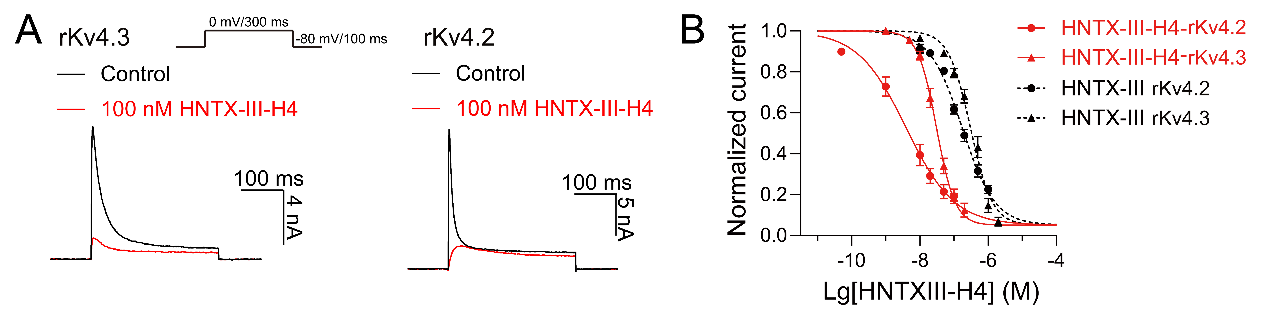


**Figure S12.** The inhibitory potency of HNTX-III-H4 on rKv4.2 or rKv4.3. (**A**) Representative current traces from HEK293T cells expressing the rKv4.2 (n=5) or rKv4.3 (n=5) in the absence (black) and presence (red) of 100 nM HNTX-III-H4. The inset shows the currents elicited protocol. (**B**) Concentration-dependent inhibitory curves show the effects of HNTX-III on rKv4.2 (n = 6) and rKv4.3 (n = 5) or HNTX-III-H4 on rKv4.2 (n = 5) and rKv4.3 (n = 5). Data are presented as mean ± S.E.M.


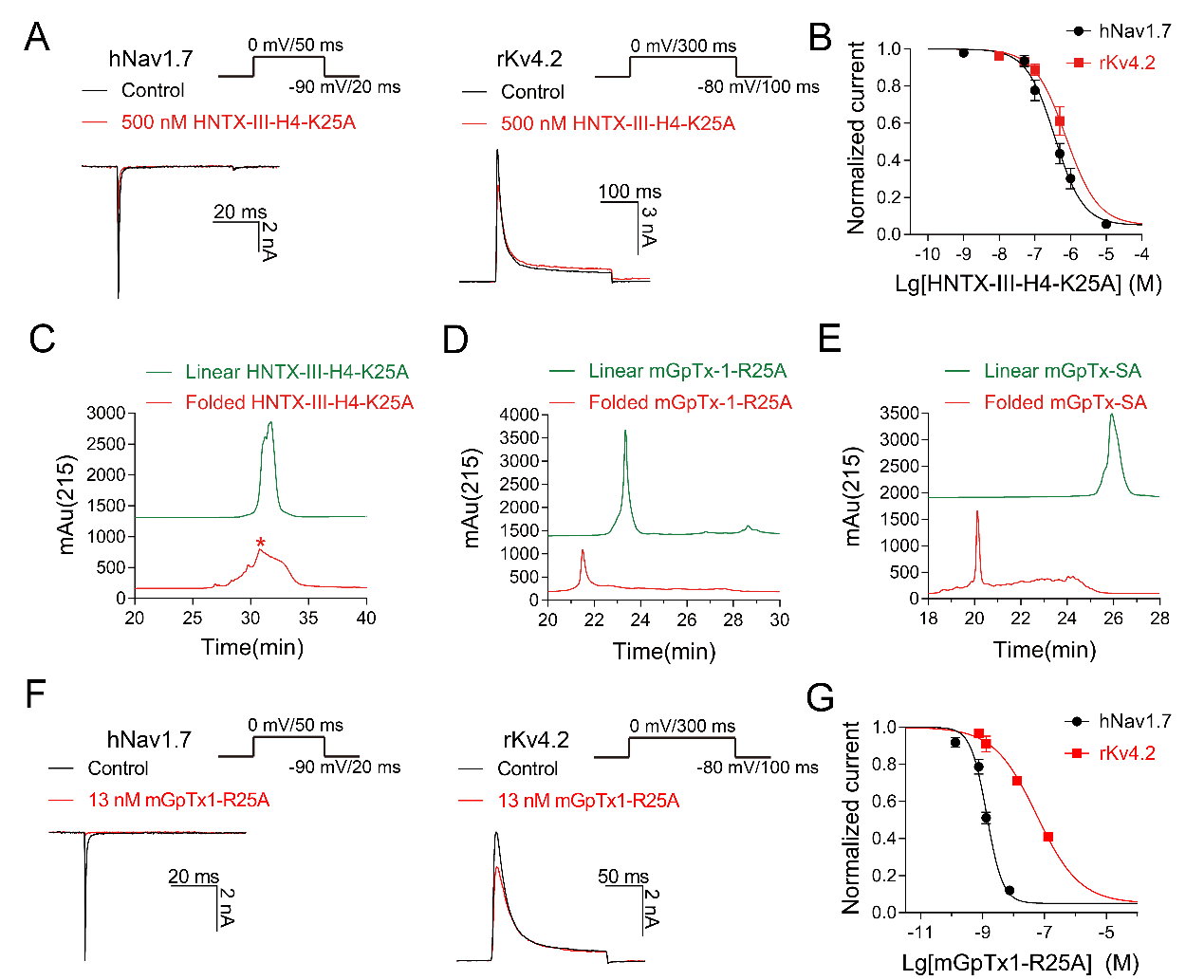


**Figure S13.** The inhibitory potency and selectivity of HNTX-III-H4 analogue H4-K25A and mGpTx1 analogue mGpTx-1-R25A. (**A**) Representative current traces from HEK293T cells expressing the rKv4.2 or hNav1.7 in the absence (black) and presence (red) of 500 nM H4-K25A. The inset shows the currents elicited protocol. (**B**) Concentration-dependent inhibitory curves show the effects of H4-K25A on hNav1.7 (black, n = 3) or rKv4.2 (red, n = 3). (**C**) RP-HPLC chromatograms of crude linear H4-K25A (top, green), and refolded H4-K25A (bottom, red). (The target fraction is marked with *). (**D**) RP-HPLC chromatograms of crude linear mGpTx-1-R25A (top, green), and refolded mGpTx-1-R25A (bottom, red) (**E**) RP-HPLC chromatograms of crude linear mGpTx-1-SA (top, green), and refolded mGpTx-1-SA (bottom, red). (**F**) Representative current traces from HEK293T cells expressing the rKv4.2 or hNav1.7 in the absence (black) and presence (red) of 13 nM mGpTx1-R25A. The inset shows the currents elicited protocol. (**G**) Concentration-dependent inhibitory curves show the effects of mGpTx1-R25A on hNav1.7 (black, n = 4) or rKv4.2 (red, n = 3). Data are presented as mean ± S.E.M.


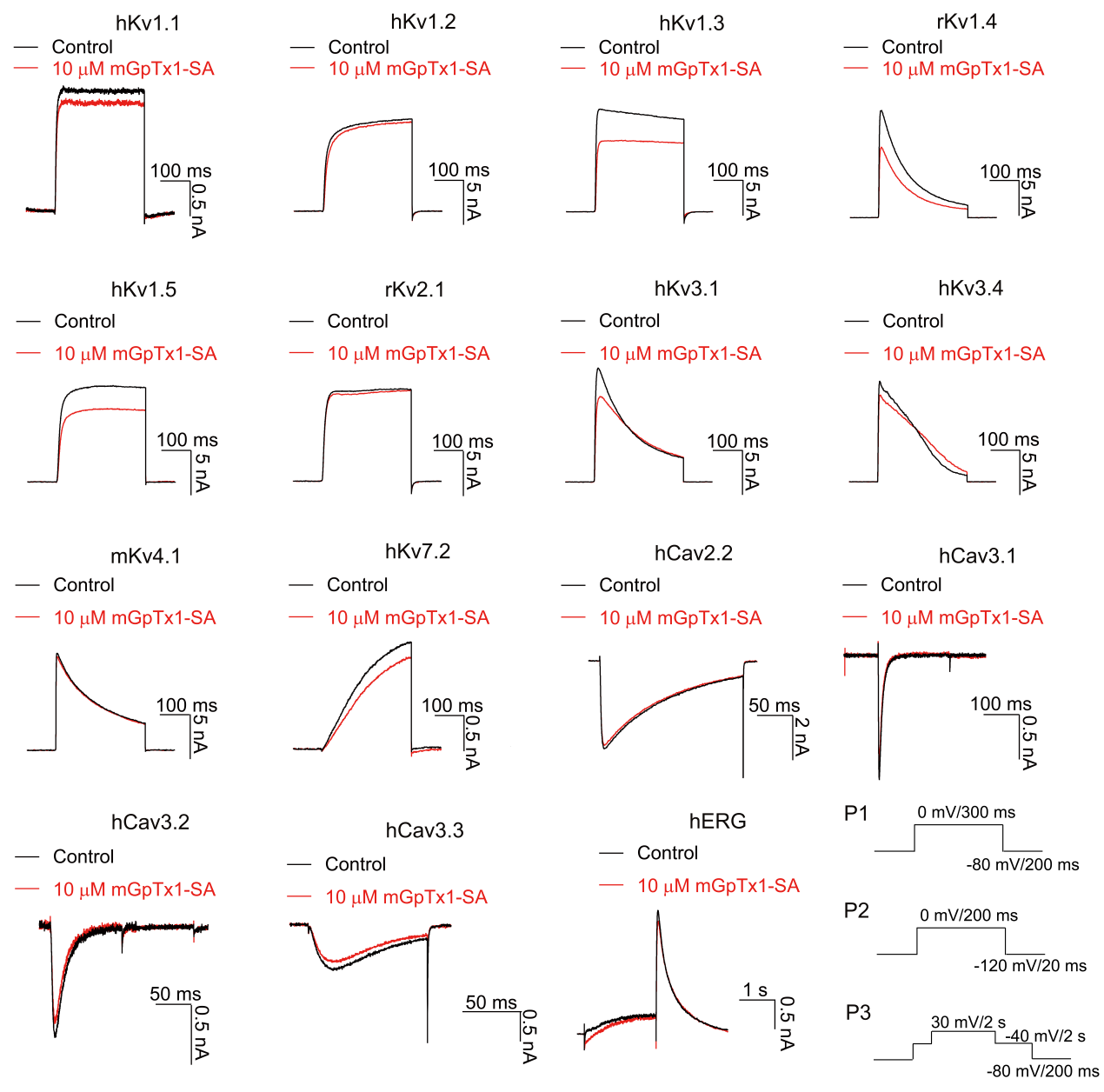


**Figure S14.** The activity of mGpTx1-SA on Kv and Cav subtypes. Representative current traces from HEK293T cells or CHO cells expressing hKv1.1-1.5, rKv2.1, hKv3.1-3.4, mKv4.1, hKv7.2 and hERG or hCav2.2, hCav3.-3.3 in the absence (black) or presence (red) of 10 μM mGpTx1-SA (n = 3-4). The currents of Kv channels, Cav channels, and hERG were elicited by protocol P1, P2 and P3, respectively.


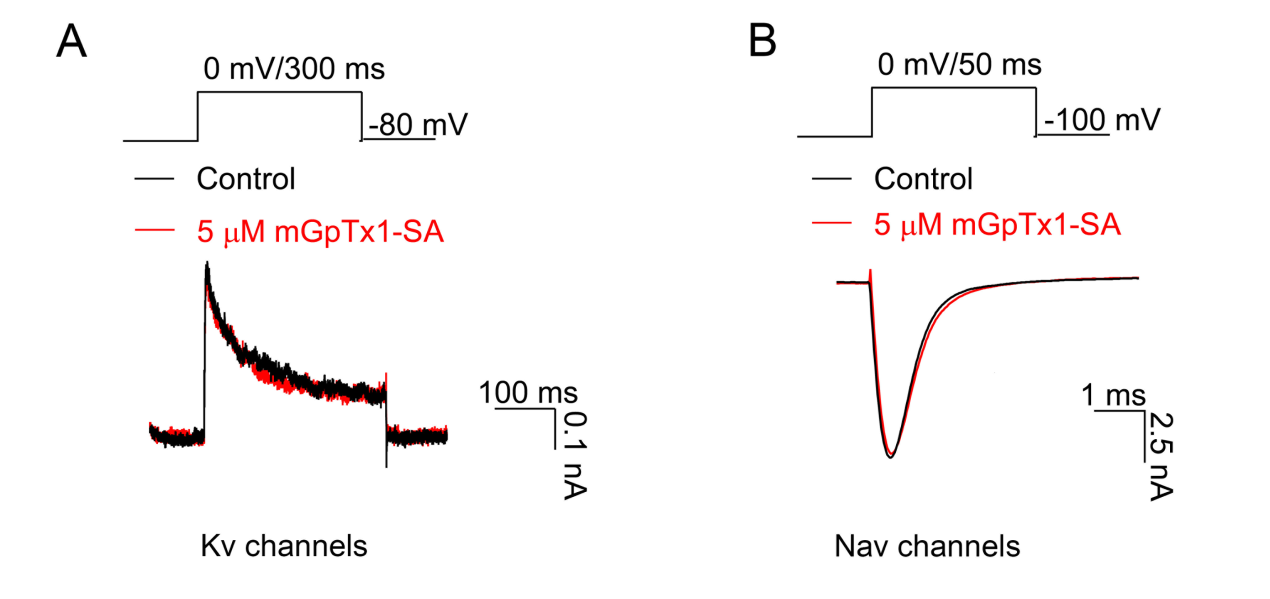


**Figure S15.** The effect of mGpTx1-SA on native sodium and potassium-mediated currents of primary cardiomyocytes. (**A** and **B**) Representative current traces from rat primary cardiomyocytes expressing native Kv channels (**A**) or native Nav channels (**B**) treated by 5 μM mGpTx1-SA (red) respectively (n = 5). The upper panel shows the currents elicited protocol.


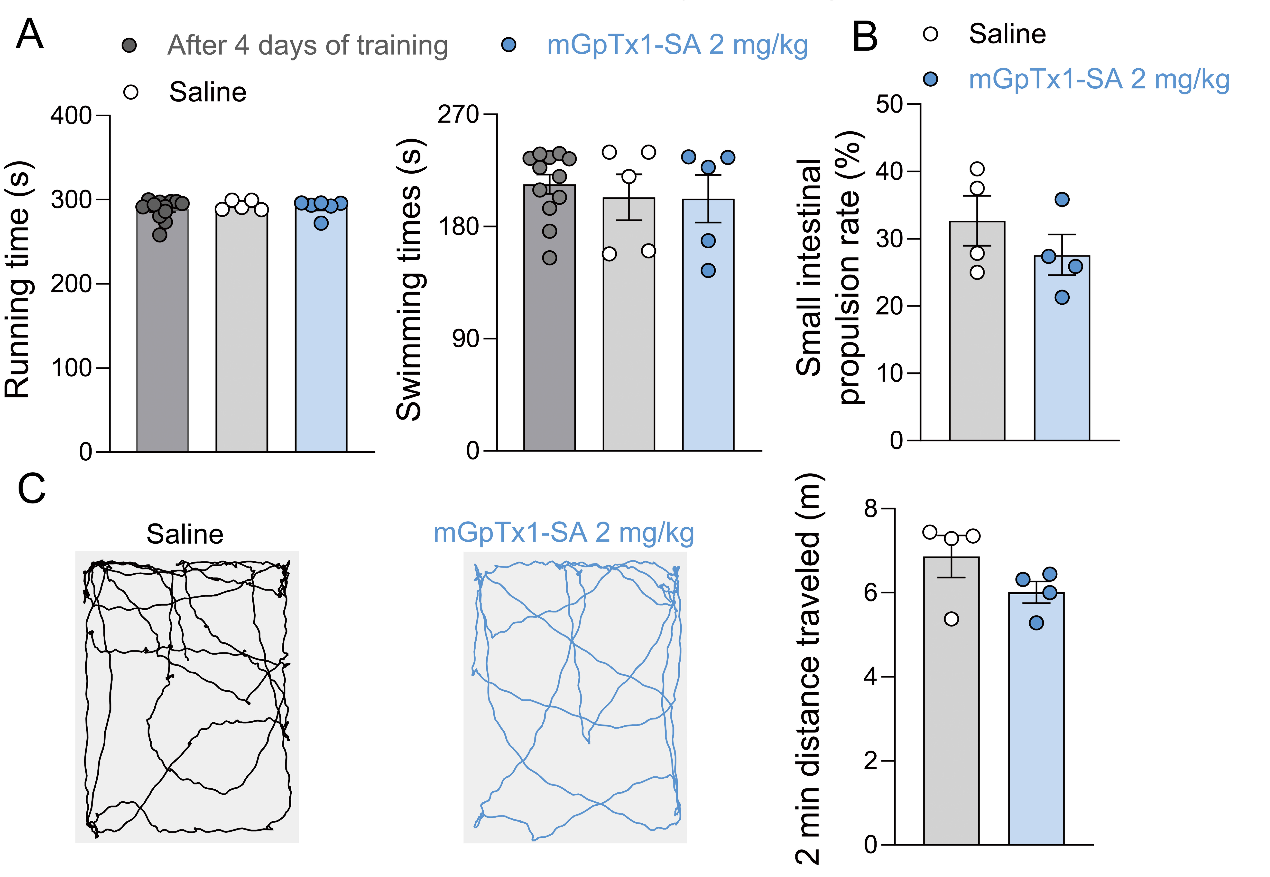


**Figure S16.** *In vivo* safety evaluation of mGpTx1-SA. (**A**) Histograms show the running time and swimming time of mice, which were trained for four days (gray) and then treated by saline (white, n = 5) or 2 mg/kg mGpTx1-SA (blue, n = 5-6) with i.p. (followed by no paired t test). (**B**) Small intestinal propulsion rate of ICR mice treated by saline (white, n=4) or 2 mg/kg mGpTx1-SA (blue, n=4) with i.p. (followed by no paired t test). (**C**) Shown on the right is the trajectories of the mice and the left is the distance traveled of mice, with treated by saline (white, n=4) or 2 mg/kg mGpTx1-SA (blue, n=4) with i.p. (followed by no paired t test). Data are represented as mean ± S.E.M.

**Supplementary Tables**

**Supplementary Table S1.** The Hill coefficients for the eight toxins on rKv4.2

| Toxins | Hill coefficients | n |
| --- | --- | --- |
| HNTX-III | 0.93 ± 0.08 | 6 |
| mGpTx1 | 0.77 ± 0.07 | 6 |
| ProTx-II | 0.72 ± 0.04 | 4 |
| HWTX-IV | 1.02 ± 0.07 | 8 |
| Cl6b | 1.03 ± 0.20 | 4 |
| Ca2a | 1.05 ± 0.17 | 5 |
| HNTX-IV | 1.30 ± 0.31 | 5 |
| HNTX-VIIa | 2.4 ± 0.3 | 4 |

Data are represented as mean ± S.E.M

**Supplementary Table S2.** The summary of the eight toxins affecting the steady-state activation and inactivation of rKv4.2.

|  | Voltage Dependence of activation (mV) | | | | | | | | | Voltage Dependence of inactivation (mV) | | | | |  |
| --- | --- | --- | --- | --- | --- | --- | --- | --- | --- | --- | --- | --- | --- | --- | --- |
| Toxins | Control | | | | Toxins^#^ | | |  | Control | | | Toxins^#^ | | |  |
|  | V_1/2_ | κ | n | V_1/2_ | | κ | | n | V_1/2_ | | κ | n | V_1/2_ | κ | n |
| HNTX-III | -3.9±4.3 | 30.5±1.7 | 7 | 26.3±5.1^***^ | | | 38.2±3.3 | 7 | -54.1±3.4 | | 7.7±0.4 | 7 | -48.0±3.7 | 7.4±0.6 | 7 |
| mGpTx1 | -3.0±1.9 | 21.4±1.5 | 7 | 23.8±1.7^***^ | | | 27.2±2.7 | 7 | -53.6±1.1 | | 6.3±0.6 | 9 | -41.7±2.0^***^ | 5.6±0.3 | 9 |
| ProTx-II | -8.7±1.9 | 15.3±0.6 | 8 | 31.8±2.0^***^ | | | 17.6±0.7 | 8 | -57.1±1.8 | | 5.6±0.3 | 7 | -27.5±2.3^***^ | 5.8±0.3 | 7 |
| HWTX-IV | -1.0±6.3 | 25.6±2.5 | 7 | 24.6±6.3^*^ | | | 23.5±1.6 | 7 | -59.4±3.1 | | 8.3±1.2 | 7 | -39.4±3.2^**^ | 8.5±1.2 | 7 |
| Cl6b | 24.9±7.2 | 24.4±2.3 | 6 | 52.4±9.1^****^ | | | 20.3±1.4 | 6 | -59.1±4.1 | | 6.3±0.6 | 6 | -48.8±3.3^*^ | 7.7±0.9 | 6 |
| Ca2a | 12.0±2.4 | 24.4±2.3 | 6 | 42.0±2.1^****^ | | | 20.3±1.4 | 6 | -59.9±0.9 | | 6.3±0.6 | 5 | -54.3±1.4^*^ | 7.7±0.9 | 5 |
| HNTX-IV | 8.7±1.5 | 22.8±2.2 | 6 | 29.1±2.2^****^ | | | 17.1±0.7 | 6 | -51.6±1.8 | | 5.4±0.3 | 6 | -46.6±3.0^*^ | 5.7±0.3 | 6 |
| HNTX-VIIa | 14.4±1.8 | 23.6±1.3 | 9 | 44.3±1.6^***^ | | | 15.9±1.1 | 9 | -47.2±3.3 | | 6.3±0.4 | 7 | -16.9±5.5^*^ | 8.6±0.7 | 7 |

^#^The toxin concentrations used for HNTX-III, mGpTx1, ProTx-II, HWTX-IV, Cl6b, Ca2a, HNTX-IV and HNTX-VIIa were 200 nM, 100 nM, 10 nM, 200 nM, 200 nM, 500 nM, 100 nM and 50 nM, respectively. Data are represented as mean ± S.E.M. ^*^*p* < 0.05, ^**^*p* < 0.01, ^***^*p* < 0.001, ^****^*p* < 0.0001 versus Control (followed by paired t test).

**Supplementary Table S3.** The effects of the eight toxins on the steady-state activation and steady-state inactivation of the rKv4.3 channel

|  | Voltage dependence of activation (mV) | | | | | | | Voltage dependence of inactivation (mV) | | | | | | |  |
| --- | --- | --- | --- | --- | --- | --- | --- | --- | --- | --- | --- | --- | --- | --- | --- |
| Toxins | Control | | | | Toxins^#^ | |  | Control | | Toxins^#^ | | | | |  |
|  | V_1/2_ | κ | n | V_1/2_ | | κ | n | V_1/2_ | κ | n | | V_1/2_ | κ | | n |
| HNTX-III | -4.1±1.4 | 20.8±1.5 | 9 | 25.1±4.6^****^ | | 23.9±2.4 | 9 | -50.2±1.9 | 5.6±0.3 | 10 | -43.6±2.4^*^ | | | 7.4±0.4 | 10 |
| mGpTx1 | -7.8±1.5 | 14.5±1.3 | 8 | 59.6±2.1^***^ | | 25.3±1.3 | 8 | -47.0±1.3 | 5.3±0.3 | 9 | -27.7±3.0^***^ | | | 9.3±0.6 | 9 |
| ProTx-II | -9.0±4.9 | 20.7±1.0 | 8 | 33.4±6.6^***^ | | 28.4±2.8 | 8 | -49.4±1.4 | 5.8±0.4 | 5 | -37.5±2.1^**^ | | | 7.6±0.5 | 5 |
| HWTX-IV | -5.7±1.6 | 20.3±1.1 | 8 | 42.6±3.5^***^ | | 33.5±1.7 | 8 | -47.2±2.0 | 5.7±0.3 | 7 | -38.4±1.7^*^ | | | 7.4±0.3 | 7 |
| Cl6b | 16.7±1.9 | 21.4±1.0 | 7 | 34.8±1.4^****^ | | 21.2±0.7 | 7 | -49.9±2.3 | 6.4±0.6 | 8 | -45.1±1.8^**^ | | | 6.5±0.2 | 8 |
| Ca2a | 12.8±1.5 | 26.8±0.9 | 5 | 59.0±3.0^****^ | | 12.2±0.9 | 5 | -48.4±1.0 | 5.0±0.2 | 7 | -48.1±2.0 | | | 4.5±0.2 | 7 |
| HNTX-IV | 9.3±2.2 | 29.3±1.3 | 6 | 67.9±3.2^****^ | | 18.4±2.0 | 6 | -50.9±1.2 | 4.7±0.2 | 6 | -41.7±1.5^**^ | | | 6.3±0.3 | 6 |
| HNTX-VIIa | 14.3±3.7 | 29.2±1.6 | 5 | 61.7±2.5^***^ | | 17.4±1.9 | 5 | -51.3±0.9 | 4.3±0.1 | 6 | -44.3±1.6^**^ | | | 7.1±0.6 | 6 |

^#^The toxin concentrations used for HNTX-III, mGpTx1, ProTx-II, HWTX-IV, Cl6b, Ca2a, HNTX-IV and HNTX-VIIa were 500 nM, 100 nM, 50 nM, 500 nM, 100 nM, 500 nM, 100 nM and 100 nM, respectively. Data are represented as mean ± S.E.M. ^*^*p* < 0.05, ^**^*p* < 0.01, ^***^*p* < 0.001, ^****^*p* < 0.0001 versus Control (followed by pair t test).

**Supplementary Table S4.** The IC_50_ values of HNTX-III on rKv4.2 mutants

| Compounds | Substitution | HNTX-III（μM） | n |
| --- | --- | --- | --- |
| 1 | rKv4.2 WT | 0.162 ± 0.023 | 6 |
| 2 | V278A | 0.177 ± 0.063 | 4 |
| 3 | M279A | 1.000 ± 0.347 | 4 |
| 4 | T280A | 0.038 ± 0.014 | 4 |
| 5 | D281A | 0.214 ± 0.076 | 4 |
| 6 | D281K | 5.000 ± 1.000 | 3 |
| 7 | D281N | 0.131 ± 0.032 | 4 |
| 8 | N282A | 0.008 ± 0.002 | 4 |
| 9 | E283A | 0.987 ± 0.303 | 4 |
| 10 | E283K | 3.000 ± 1.000 | 4 |
| 11 | D284A | 0.491 ± 0.072 | 4 |
| 12 | D284K | >20 | 3 |

IC_50_ values are expressed as mean ± SEM, n = 3-6.

**Supplementary Table S5.** The IC_50_ values of mGxpTx1 on rKv4.2 mutants

| Compounds | Substitution | mGpTx1（μM） | n |
| --- | --- | --- | --- |
| 1 | rKv4.2 WT | 0.081 ± 0.016 | 6 |
| 2 | V278A | 2.848 ± 1.309 | 5 |
| 3 | M279A | 16.514 ± 9.006 | 5 |
| 4 | T280A | 0.150 ± 0.032 | 5 |
| 5 | D281K | 0.320 ± 0.045 | 6 |
| 6 | N282A | 0.017 ± 0.005 | 5 |
| 7 | E283K | 0.646 ± 0.135 | 7 |
| 8 | D284K | 0.663 ± 0.179 | 5 |

IC_50_ values are expressed as mean ± SEM, n = 5-7.

**Supplementary Table S6.** The IC_50_ values of ProTx-II on rKv4.2 mutants

| Compounds | Substitution | ProTx-II（μM） | n |
| --- | --- | --- | --- |
| 1 | rKv4.2 WT | 0.005 ± 0.002 | 4 |
| 2 | V278A | 0.010 ± 0.002 | 5 |
| 3 | M279A | 0.017 ± 0.005 | 5 |
| 4 | T280A | 0.089 ± 0.021 | 5 |
| 5 | D281K | 0.027 ± 0.002 | 5 |
| 6 | N282A | 0.007 ± 0.001 | 6 |
| 7 | E283K | 0.014 ± 0.006 | 7 |
| 8 | D284K | 0.020 ± 0.002 | 5 |

IC_50_ values are expressed as mean ± SEM, n = 4-7.

**Supplementary Table S7.** The IC_50_ values of HWTX-IV on rKv4.2 mutants

| Compounds | Substitution | HWTX-IV（μM） | n | |
| --- | --- | --- | --- | --- |
| 1 | rKv4.2 WT | 0.058 ± 0.011 | 8 |  |
| 2 | V278A | 0.222 ± 0.057 | 5 |  |
| 3 | M279A | 1.312 ± 0.271 | 5 |  |
| 4 | T280A | 0.329 ± 0.047 | 5 |  |
| 5 | D281K | 1.240 ± 0.190 | 5 |  |
| 6 | N282A | 0.012 ± 0.003 | 5 |  |
| 7 | E283K | 0.349 ± 0.089 | 6 |  |
| 8 | D284K | 2.508 ± 0.809 | 5 |  |

IC_50_ values are expressed as mean ± SEM, n = 5-8.

**Supplementary Table S8.** The IC_50_ values of Cl6b on rKv4.2 mutants

| Compounds | Substitution | Cl6b（μM） | n |
| --- | --- | --- | --- |
| 1 | rKv4.2 WT | 0.114 ± 0.032 | 4 |
| 2 | V278A | 0.145 ± 0.020 | 4 |
| 3 | M279A | 0.309 ± 0.126 | 5 |
| 4 | T280A | 0.011 ± 0.001 | 4 |
| 5 | D281K | 3.268 ± 0.361 | 3 |
| 6 | N282A | 0.003 ± 0.001 | 4 |
| 7 | E283K | 1.641 ± 0.386 | 4 |
| 8 | D284K | 15.425 ± 6.162 | 3 |

IC_50_ values are expressed as mean ± SEM, n = 3-4.

**Supplementary Table S9.** The IC_50_ values of Ca2a on rKv4.2 mutants

| Compounds | Substitution | Ca2a（μM） | n |
| --- | --- | --- | --- |
| 1 | rKv4.2 WT | 0.268 ± 0.044 | 5 |
| 2 | V278A | 2.216 ± 0.491 | 5 |
| 3 | M279A | 3.430 ± 0.430 | 4 |
| 4 | T280A | 0.601 ± 0.135 | 5 |
| 5 | D281K | 0.486 ± 0.086 | 5 |
| 6 | N282A | 0.013 ± 0.002 | 4 |
| 7 | E283K | >20 | 3 |
| 8 | D284K | >20 | 3 |

IC_50_ values are expressed as mean ± SEM, n = 3-5.

**Supplementary Table S10.** The IC_50_ values of HNTX-IV on rKv4.2 mutants

| Compounds | Substitution | HNTX-IV（μM） | n |
| --- | --- | --- | --- |
| 1 | rKv4.2 WT | 0.051 ± 0.010 | 5 |
| 2 | V278A | 0.182 ± 0.050 | 6 |
| 3 | M279A | 0.167 ± 0.014 | 6 |
| 4 | T280A | 0.136 ± 0.021 | 5 |
| 5 | D281K | 0.191 ± 0.032 | 6 |
| 6 | N282A | 0.011 ± 0.002 | 6 |
| 7 | E283K | 5.626 ± 1.283 | 4 |
| 8 | D284K | >20 | 3 |

IC_50_ values are expressed as mean ± SEM, n = 3-6.

**Supplementary Table S11.** The IC_50_ values of HNTX-VIIa on rKv4.2 mutants

| Compounds | Substitution | HNTX-VIIa（μM） | n |
| --- | --- | --- | --- |
| 1 | rKv4.2 WT | 0.018 ± 0.003 | 4 |
| 2 | V278A | 0.053 ± 0.006 | 5 |
| 3 | M279A | 0.042 ± 0.006 | 6 |
| 4 | T280A | 0.013 ± 0.002 | 7 |
| 5 | D281K | 0.033 ± 0.006 | 6 |
| 6 | N282A | 0.021 ± 0.001 | 7 |
| 7 | E283K | 0.062 ± 0.013 | 5 |
| 8 | D284K | 0.047 ± 0.006 | 7 |

IC_50_ values are expressed as mean ± SEM, n = 4-7.

**Supplementary Table S12.** The IC_50_ values of HNTX-III mutants on rKv4.2 and hNav1.7

| Compounds | Substitutions | rKv4.2（μM） | n | hNav1.7 (μM) |
| --- | --- | --- | --- | --- |
| 1 | HNTX-III-WT | 0.162 ± 0.023 | 6 | 0.207 ± 0.057 |
| 2 | G1F | 0.176 ± 0.047 | 6 | 0.282 ± 0.056^#^ |
| 3 | F5W | 0.833 ± 0.081 | 4 | 1.227 ± 0.167^#^ |
| 4 | F5L | 10.000 ± 4.000 | 4 | 2.041 ± 0.015^#^ |
| 5 | T10E | 0.452 ± 0.221 | 7 | 1.657 ± 0.037^#^ |
| 6 | G12E | 0.359 ± 0.082 | 8 | 1.891 ± 0.142^#^ |
| 7 | P18K | 0.167 ± 0.117 | 6 | 0.050 ± 0.002^#^ |
| 8 | P18A | 0.140 ± 0.043 | 7 | *nd* |
| 9 | N19A | 0.064 ± 0.009 | 7 | 0.116 ± 0.009 |
| 10 | N19K | 0.475 ± 0.103 | 5 | 0.431 ± 0.058^#^ |
| 11 | N19L | 0.042 ± 0.004 | 6 | 0.092 ± 0.046^#^ |
| 12 | Y20W | 0.228 ± 0.033 | 6 | 0.495 ± 0.039 |
| 13 | Y20A | 4.000 ± 2.000 | 6 | 7.162 ± 0.528 |
| 14 | A21L | 0.060 ± 0.010 | 6 | 0.133 ± 0.010^#^ |
| 15 | A21V | 1.000 ± 0.144 | 6 | 0.099 ± 0.019^#^ |
| 16 | K25A | >20 | 3 | 2.000 ± 0.332 |
| 17 | K25R | 0.946 ± 0.154 | 8 | 0.647 ± 0.046^#^ |
| 18 | H26L | 3.000 ± 0.632 | 4 | 5.781 ± 0.117^#^ |
| 19 | K27Q | 0.622 ± 0.138 | 4 | 0.568 ± 0.042^#^ |
| 20 | W28A | >10 | 3 | >10 |
| 21 | K30A | >10 | 3 | >10 |
| 22 | Y32A | 1.000 ± 0.177 | 6 | 0.936 ± 0.123 |
| 23 | Y32L | 0.441 ± 0.074 | 7 | 3.076 ± 0.054^#^ |
| 24 | Y32W | 0.277 ± 0.043 | 6 | 0.075 ± 0.016^#^ |
| 25 | L33F | 0.148 ± 0.017 | 8 | 0.215 ± 0.029^#^ |
| 26 | L33K | 20.000 ± 4.000 | 5 | >10 |

IC_50_ values are expressed as mean ± SEM, n = 3-8. "^#^" indicates the values are quoted from experimental results published in our previous studies ^[11c]^. *nd* means the data is unknown.
